# Erratum for Green et al., “Isolation and characterization of mollicute symbionts from a fungus-growing ant reveals high niche overlap leading to co-exclusion”

**DOI:** 10.1128/mbio.00225-26

**Published:** 2026-02-10

**Authors:** Emily A. Green, Ian Klepacki, Jonathan L. Klassen

## ERRATUM

Volume 16, no. 7, e00893-25, 2025, https://doi.org/10.1128/mbio.00893-25. Discussion section, “Description of *Mesoplasma whartonense*,” sentences 1 and 2: “Wharton State Park” should read “Wharton State Forest.”

Discussion section, “Description of *Spiroplasma attinicola*,” sentence 2: “Wharton State Park” should read “Wharton State Forest.”

Discussion section, “Description of *Spiroplasma attinicola*,” last sentence: “NCTC 14964” should read “NCTC 14864.”

