# Supplementary material for: Isolation and characterization of mollicute symbionts from a fungus-growing ant reveals high niche overlap leading to co-exclusion
Source: mBio. 2025 Jun 10;16(7):e00893-25. doi: 10.1128/mbio.00893-25 (PMC12239587; doi:10.1128/mbio.00893-25)
Supplement: Supplemental Code — All code needed to recapitulate the analyses in the paper. [file mbio.00893-25-s0001.docx]

**Supplementary Code**

All code needed to reproduce the analyses in Green et al. 2025 are described as follows. Note that this description assumes certain input file and working directory names. However, it includes all parameters used so that all analyses can be recapitulated faithfully.

#Run fastqc

mkdir MesoFastqc

fastqc ~/MollicuteGenomes2021/*.fastq -o ~/MollicuteGenomes2021/MesoFastqc/

#Need to remove adapters, use trimmomatic

#MUST MOVE ADAPTER FILE TO DIRECTORY YOU WORK IN

cp /usr/local/bioinf_tools/Trimmomatic-0.39/adapters/TruSeq2-PE.fa ~/MollicuteGenomes2021/

java -jar /usr/local/bioinf_tools/Trimmomatic-0.39/trimmomatic-0.39.jar PE -threads 8 -phred33 JKS002656_S195_L001_R1_001.fastq JKS002656_S195_L001_R2_001.fastq JKS002656_R1_paired.fastq JKS002656_R1_unpaired.fastq JKS002656_R2_paired.fastq JKS002656_R2_upaired.fastq ILLUMINACLIP:TruSeq2-PE.fa:2:30:10 LEADING:3 TRAILING:3 SLIDINGWINDOW:4:15 MINLEN:36

java -jar /usr/local/bioinf_tools/Trimmomatic-0.39/trimmomatic-0.39.jar PE -threads 8 -phred33 JKS002657_S196_L001_R1_001.fastq JKS002657_S196_L001_R2_001.fastq JKS002657_R1_paired.fastq JKS002657_R1_unpaired.fastq JKS002657_R2_paired.fastq JKS002657_R2_unpaired.fastq ILLUMINACLIP:TruSeq2-PE.fa:2:30:10 LEADING:3 TRAILING:3 SLIDINGWINDOW:4:15 MINLEN:36

java -jar /usr/local/bioinf_tools/Trimmomatic-0.39/trimmomatic-0.39.jar PE -threads 8 -phred33 JKS002658_S197_L001_R1_001.fastq JKS002658_S197_L001_R2_001.fastq JKS002658_R1_paired.fastq JKS002658_R1_unpaired.fastq JKS002658_R2_paired.fastq JKS002658_R2_unpaired.fastq ILLUMINACLIP:TruSeq2-PE.fa:2:30:10 LEADING:3 TRAILING:3 SLIDINGWINDOW:4:15 MINLEN:36

java -jar /usr/local/bioinf_tools/Trimmomatic-0.39/trimmomatic-0.39.jar PE -threads 8 -phred33 JKS002659_S198_L001_R1_001.fastq JKS002659_S198_L001_R2_001.fastq JKS002659_R1_paired.fastq JKS002659_R1_unpaired.fastq JKS002659_R2_paired.fastq JKS002659_R2_unpaired.fastq ILLUMINACLIP:TruSeq2-PE.fa:2:30:10 LEADING:3 TRAILING:3 SLIDINGWINDOW:4:15 MINLEN:36

#Run Fastqc

fastqc *_paired.fastq -o MesoFastqc

#Downloaded libraries that were rerun (JKS002660-2664) on 6-13-2021

mkdir ReRanLibraryCheck

#Run fastqc

mkdir REMesoFastqc

fastqc ~/MollicuteGenomes2021/ReRanLibraryCheck/*.fastq -o ~/MollicuteGenomes2021/ReRanLibraryCheck/REMesoFastqc/

#Need to remove adapters, use trimmomatic

#MUST MOVE ADAPTER FILE TO DIRECTORY YOU WORK IN

cp /usr/local/bioinf_tools/Trimmomatic-0.39/adapters/TruSeq2-PE.fa ~/MollicuteGenomes2021/ReRanLibraryCheck

java -jar /usr/local/bioinf_tools/Trimmomatic-0.39/trimmomatic-0.39.jar PE -threads 8 -phred33 JKS002660_S143_L001_R1_001.fastq JKS002660_S143_L001_R2_001.fastq JKS002660_R1_paired.fastq JKS002660_R1_unpaired.fastq JKS002660_R2_paired.fastq JKS002660_R2_unpaired.fastq ILLUMINACLIP:TruSeq2-PE.fa:2:30:10 LEADING:3 TRAILING:3 SLIDINGWINDOW:4:15 MINLEN:36

java -jar /usr/local/bioinf_tools/Trimmomatic-0.39/trimmomatic-0.39.jar PE -threads 8 -phred33 JKS002661_S144_L001_R1_001.fastq JKS002661_S144_L001_R2_001.fastq JKS002661_R1_paired.fastq JKS002661_R1_unpaired.fastq JKS002661_R2_paired.fastq JKS002661_R2_unpaired.fastq ILLUMINACLIP:TruSeq2-PE.fa:2:30:10 LEADING:3 TRAILING:3 SLIDINGWINDOW:4:15 MINLEN:36

java -jar /usr/local/bioinf_tools/Trimmomatic-0.39/trimmomatic-0.39.jar PE -threads 8 -phred33 JKS002662_S145_L001_R1_001.fastq JKS002662_S145_L001_R2_001.fastq JKS002662_R1_paired.fastq JKS002662_R1_unpaired.fastq JKS002662_R2_paired.fastq JKS002662_R2_unpaired.fastq ILLUMINACLIP:TruSeq2-PE.fa:2:30:10 LEADING:3 TRAILING:3 SLIDINGWINDOW:4:15 MINLEN:36

java -jar /usr/local/bioinf_tools/Trimmomatic-0.39/trimmomatic-0.39.jar PE -threads 8 -phred33 JKS002663_S146_L001_R1_001.fastq JKS002663_S146_L001_R2_001.fastq JKS002663_R1_paired.fastq JKS002663_R1_unpaired.fastq JKS002663_R2_paired.fastq JKS002663_R2_unpaired.fastq ILLUMINACLIP:TruSeq2-PE.fa:2:30:10 LEADING:3 TRAILING:3 SLIDINGWINDOW:4:15 MINLEN:36

java -jar /usr/local/bioinf_tools/Trimmomatic-0.39/trimmomatic-0.39.jar PE -threads 8 -phred33 JKS002664_S147_L001_R1_001.fastq JKS002664_S147_L001_R2_001.fastq JKS002664_R1_paired.fastq JKS002664_R1_unpaired.fastq JKS002664_R2_paired.fastq JKS002664_R2_unpaired.fastq ILLUMINACLIP:TruSeq2-PE.fa:2:30:10 LEADING:3 TRAILING:3 SLIDINGWINDOW:4:15 MINLEN:36

#Run Fastqc

fastqc *_paired.fastq -o REMesoFastqc

#unicycler

unicycler -1 JKS002660_R1_paired.fastq -2 JKS002660_R2_paired.fastq -o JKS002660_unicycler -t 20

unicycler -1 JKS002661_R1_paired.fastq -2 JKS002661_R2_paired.fastq -o JKS002661_unicycler -t 20

unicycler -1 JKS002662_R1_paired.fastq -2 JKS002662_R2_paired.fastq -o JKS002662_unicycler -t 20

unicycler -1 JKS002663_R1_paired.fastq -2 JKS002663_R2_paired.fastq -o JKS002663_unicycler -t 22

unicycler -1 JKS002664_R1_paired.fastq -2 JKS002664_R2_paired.fastq -o JKS002664_unicycler -t 22

#check unicycler assembly

quast.py ~/MollicuteGenomes2021/ReRanLibraryCheck/JKS002660_unicycler/JKS002660_assembly.fasta -o ~/MollicuteGenomes2021/ReRanLibraryCheck/QuastOutput/JKS002660_unicycler

quast.py ~/MollicuteGenomes2021/ReRanLibraryCheck/JKS002661_unicycler/JKS002661_assembly.fasta -o ~/MollicuteGenomes2021/ReRanLibraryCheck/QuastOutput/JKS002661_unicycler

quast.py ~/MollicuteGenomes2021/ReRanLibraryCheck/JKS002662_unicycler/JKS002662_assembly.fasta -o ~/MollicuteGenomes2021/ReRanLibraryCheck/QuastOutput/JKS002662_unicycler

quast.py ~/MollicuteGenomes2021/ReRanLibraryCheck/JKS002663_unicycler/JKS002663_assembly.fasta -o ~/MollicuteGenomes2021/ReRanLibraryCheck/QuastOutput/JKS002663_unicycler

quast.py ~/MollicuteGenomes2021/ReRanLibraryCheck/JKS002664_unicycler/JKS002664_assembly.fasta -o ~/MollicuteGenomes2021/ReRanLibraryCheck/QuastOutput/JKS002664_unicycler

#Run Unicycler assemblies for the other genomes (JKS002656-2659)

unicycler -1 JKS002656_R1_paired.fastq -2 JKS002656_R2_paired.fastq -o JKS002656_unicycler -t 22

unicycler -1 JKS002657_R1_paired.fastq -2 JKS002657_R2_paired.fastq -o JKS002657_unicycler -t 22

unicycler -1 JKS002658_R1_paired.fastq -2 JKS002658_R2_paired.fastq -o JKS002658_unicycler -t 22

unicycler -1 JKS002659_R1_paired.fastq -2 JKS002659_R2_paired.fastq -o JKS002659_unicycler -t 22

#Check w quast

quast.py ~/MollicuteGenomes2021/JKS002656_unicycler/JKS002656_assembly.fasta -o ~/MollicuteGenomes2021/QuastOutput/JKS002656_unicycler

quast.py ~/MollicuteGenomes2021/JKS002657_unicycler/JKS002657_assembly.fasta -o ~/MollicuteGenomes2021/QuastOutput/JKS002657_unicycler

quast.py ~/MollicuteGenomes2021/JKS002658_unicycler/JKS002658_assembly.fasta -o ~/MollicuteGenomes2021/QuastOutput/JKS002658_unicycler

quast.py ~/MollicuteGenomes2021/JKS002659_unicycler/JKS002659_assembly.fasta -o ~/MollicuteGenomes2021/QuastOutput/JKS002659_unicycler

#Genomes JKS002657-64 are 100% complete!

https://busco.ezlab.org/busco_userguide.html to understand results

#Check the non filtered busco genomes

busco -m genome -i JKS002656_assembly.fasta -o JKS002656_busco -l /isg/shared/databases/BUSCO/odb10/lineages/tenericutes_odb10/

busco -m genome -i JKS002657_assembly.fasta -o JKS002657_busco -l /isg/shared/databases/BUSCO/odb10/lineages/tenericutes_odb10/

busco -m genome -i JKS002658_assembly.fasta -o JKS002658_busco -l /isg/shared/databases/BUSCO/odb10/lineages/tenericutes_odb10/

https://merenlab.org/2016/06/22/anvio-tutorial-v2/

#https://merenlab.org/2017/05/18/working-with-prokka/

wget https://raw.githubusercontent.com/karkman/gff_parser/master/gff_parser.py -O gff_parser.py

# pip install gffutils didn't work

conda install -c bioconda gffutils

#I need the raw reads so I can run them through unicycler. Downloaded from https://www.ncbi.nlm.nih.gov/sra/SRX3221016[accn]

#A reference mesoplasma lactucae genome by Gingko bioworks

mkdir FastqcCheck

fastqc ~/MollicuteGenomes2021/NCBIGenomeTest *.fastq -o ~/MollicuteGenomes2021/NCBIGenomeTest/FastqcCheck

unicycler -1 SRR6082029.1_1.fastq -2 SRR6082029.1_2.fastq -o SRR6082029_unicycler -t 20

quast.py ~/MollicuteGenomes2021/NCBIGenomeTest/SRR6082029_unicycler/assembly.fasta -o ~/MollicuteGenomes2021/NCBIGenomeTest/SRR6082029_quast/

#Blasted all contigs from each genome that anvio didn't find to add to the "completeness" of the genome.

#The matches are to very random things (dog, tuna, moth, etc).

#There is a ton of overlap of small contigs from each of the genomes.

#I then aligned those contigs with the Mesoplasma lactucae genome from above that is on NCBI.

#There is minimal alignment to the Meso lactu genome. ~16 to 30 bp aligning out of thecontigs that are 188 to 900 bp long.

#I will now remove those contigs from the fasta files.

#Logged onto Xanadu

mkdir MollicuteGenomes2021

busco -m genome -i FiltJKS002656_assembly.fasta -o FiltJKS002656_busco -l /isg/shared/databases/BUSCO/odb10/lineages/tenericutes_odb10/

busco -m genome -i FiltJKS002657_assembly.fasta -o FiltJKS002657_busco -l /isg/shared/databases/BUSCO/odb10/lineages/tenericutes_odb10/

busco -m genome -i FiltJKS002658_assembly.fasta -o FiltJKS002658_busco -l /isg/shared/databases/BUSCO/odb10/lineages/tenericutes_odb10/

busco -m genome -i FiltJKS002659_assembly.fasta -o FiltJKS002659_busco -l /isg/shared/databases/BUSCO/odb10/lineages/tenericutes_odb10/

busco -m genome -i FiltJKS002660_assembly.fasta -o FiltJKS002660_busco -l /isg/shared/databases/BUSCO/odb10/lineages/tenericutes_odb10/

busco -m genome -i FiltJKS002661_assembly.fasta -o FiltJKS002661_busco -l /isg/shared/databases/BUSCO/odb10/lineages/tenericutes_odb10/

busco -m genome -i FiltJKS002662_assembly.fasta -o FiltJKS002662_busco -l /isg/shared/databases/BUSCO/odb10/lineages/tenericutes_odb10/

busco -m genome -i FiltJKS002663_assembly.fasta -o FiltJKS002663_busco -l /isg/shared/databases/BUSCO/odb10/lineages/tenericutes_odb10/

busco -m genome -i FiltJKS002664_assembly.fasta -o FiltJKS002664_busco -l /isg/shared/databases/BUSCO/odb10/lineages/tenericutes_odb10/

quast.py ~/MollicuteGenomes2021/Genomes_Meso_filtered/FiltJKS002657.fasta -o ~/MollicuteGenomes2021/Genomes_Meso_filtered/QuastOutput/FiltJKS002657

quast.py ~/MollicuteGenomes2021/Genomes_Meso_filtered/FiltJKS002658.fasta -o ~/MollicuteGenomes2021/Genomes_Meso_filtered/QuastOutput/FiltJKS002658

quast.py ~/MollicuteGenomes2021/Genomes_Meso_filtered/FiltJKS002659.fasta -o ~/MollicuteGenomes2021/Genomes_Meso_filtered/QuastOutput/FiltJKS002659

quast.py ~/MollicuteGenomes2021/Genomes_Meso_filtered/FiltJKS002660.fasta -o ~/MollicuteGenomes2021/Genomes_Meso_filtered/QuastOutput/FiltJKS002660

quast.py ~/MollicuteGenomes2021/Genomes_Meso_filtered/FiltJKS002661.fasta -o ~/MollicuteGenomes2021/Genomes_Meso_filtered/QuastOutput/FiltJKS002661

quast.py ~/MollicuteGenomes2021/Genomes_Meso_filtered/FiltJKS002662.fasta -o ~/MollicuteGenomes2021/Genomes_Meso_filtered/QuastOutput/FiltJKS002662

quast.py ~/MollicuteGenomes2021/Genomes_Meso_filtered/FiltJKS002663.fasta -o ~/MollicuteGenomes2021/Genomes_Meso_filtered/QuastOutput/FiltJKS002663

quast.py ~/MollicuteGenomes2021/Genomes_Meso_filtered/FiltJKS002664.fasta -o ~/MollicuteGenomes2021/Genomes_Meso_filtered/QuastOutput/FiltJKS002664

#In JGI, click on Metagenomes by ecosystem- host-associated- fungi (right most)- search for Mollicutes

#Click on bin_id (far left column). The page that opens will have information about that Mollicute bin. Select # of scaffolds, opens page with each scaffold listed. Add scaffolds to cart and export

busco -m genome -i TSFL002_MetaG.fasta -o TSFL002_busco -l /isg/shared/databases/BUSCO/odb10/lineages/tenericutes_odb10/

busco -m genome -i TSFL042_MetaG.fasta -o TSFL042_busco -l /isg/shared/databases/BUSCO/odb10/lineages/tenericutes_odb10/

busco -m genome -i TSFL050_MetaG.fasta -o TSFL050_busco -l /isg/shared/databases/BUSCO/odb10/lineages/tenericutes_odb10

busco -m genome -i TSGA003_MetaG.fasta -o TSGA003_busco -l /isg/shared/databases/BUSCO/odb10/lineages/tenericutes_odb10

busco -m genome -i TSGA061_MetaG.fasta -o TSGA061_busco -l /isg/shared/databases/BUSCO/odb10/lineages/tenericutes_odb10

busco -m genome -i TSNC071_MetaG.fasta -o TSNC071_busco -l /isg/shared/databases/BUSCO/odb10/lineages/tenericutes_odb10

busco -m genome -i TSNJ022_MetaG.fasta -o TSNJ022_busco -l /isg/shared/databases/BUSCO/odb10/lineages/tenericutes_odb10

busco -m genome -i TSNJ026_MetaG.fasta -o TSNJ026_busco -l /isg/shared/databases/BUSCO/odb10/lineages/tenericutes_odb10

busco -m genome -i TSNJ034_MetaG.fasta -o TSNJ034_busco -l /isg/shared/databases/BUSCO/odb10/lineages/tenericutes_odb10

busco -m genome -i TSNJ035_MetaG.fasta -o TSNJ035_busco -l /isg/shared/databases/BUSCO/odb10/lineages/tenericutes_odb10

busco -m genome -i TSNJ092_MetaG.fasta -o TSNJ092_busco -l /isg/shared/databases/BUSCO/odb10/lineages/tenericutes_odb10

quast.py TSFL002_MetaG.fasta -o ~/JGIMeta/JGIQuast/TSFL002_check

quast.py TSFL042_MetaG.fasta -o ~/JGIMeta/JGIQuast/TSFL042_check

quast.py TSFL050_MetaG.fasta -o ~/JGIMeta/JGIQuast/TSFL050_check

quast.py TSGA003_MetaG.fasta -o ~/JGIMeta/JGIQuast/TSGA003_check

quast.py TSGA061_MetaG.fasta -o ~/JGIMeta/JGIQuast/TSGA061_check

quast.py TSNC071_MetaG.fasta -o ~/JGIMeta/JGIQuast/TSNC071_check

quast.py TSNJ022_MetaG.fasta -o ~/JGIMeta/JGIQuast/TSNJ022_check

quast.py TSNJ026_MetaG.fasta -o ~/JGIMeta/JGIQuast/TSNJ026_check

quast.py TSNJ034_MetaG.fasta -o ~/JGIMeta/JGIQuast/TSNJ034_check

quast.py TSNJ035_MetaG.fasta -o ~/JGIMeta/JGIQuast/TSNJ035_check

quast.py TSNJ092_MetaG.fasta -o ~/JGIMeta/JGIQuast/TSNJ092_check

#Making sure I have prokka annoations for all my genomes/metagenomes to go into Anvio. needed for the pangenome analysis

#I've removed contigs that are not needed from my Mesoplasma genomes.

#Created directory "Genomes_Meso_Filtered" for all the my Mesoplasma genomes I am moving forward with.

#Mesoplasma lactucae genome

anvi-script-reformat-fasta /home/egreen/MollicuteGenomes2021/Genomes_Meso_filtered/JKS002656_assembly.fasta -o JKS002656-fixed.fa -l 0 --simplify-names -r Keep2656tabs

anvi-gen-contigs-database -f JKS002656-fixed.fa -o JKS002656anvi.db -n anvitry2656

prokka --prefix PROKKA --outdir PROKKA_JKS002656-fixed --cpus 5 JKS002656-fixed.fa --gcode 4 --kingdom Bacteria

python3 gff_parser.py PROKKA_JKS002656-fixed/PROKKA.gff --gene-calls JKS002656gene_calls.txt --annotation JKS002656gene_annot.txt

anvi-gen-contigs-database -f JKS002656-fixed.fa -o JKS002656anvi.db --external-gene-calls JKS002656gene_calls.txt --ignore-internal-stop-codons -n anvitry2656

anvi-import-functions -c JKS002656anvi.db -i JKS002656gene_annot.txt

anvi-run-hmms -c JKS002656anvi.db

anvi-display-contigs-stats JKS002656anvi.db

#skipped ncbi cogs

#NEED TO MAKE BAM FILES WITH FIXED FASTA

bwa index ~/MollicuteGenomes2021/Genomes_Meso_filtered/JKS002656-fixed.fa

bwa mem ~/MollicuteGenomes2021/Genomes_Meso_filtered/JKS002656-fixed.fa ~/MollicuteGenomes2021/JKS002656_R1_paired.fastq ~/MollicuteGenomes2021/JKS002656_R2_paired.fastq > JKS002656Aligned.sam -t 10

samtools index JKS002656Aligned.sam

samtools fixmate -O bam JKS002656Aligned.sam JKS002656Aligned_fixmate.bam

samtools sort -o JKS002656Aligned_sorted.bam -O bam -T temp JKS002656Aligned_fixmate.bam

samtools index JKS002656Aligned_sorted.bam

anvi-init-bam JKS002656Aligned_sorted.bam -o JKS002656.bam

anvi-run-scg-taxonomy -c JKS002656anvi.db

anvi-profile -i JKS002656.bam -c JKS002656anvi.db --num-threads 10 --output-dir JKS002656_AnviProf --min-contig-length 100 --sample-name JKS002656 --cluster-contigs

anvi-interactive -p ~/MollicuteGenomes2021/Genomes_Meso_filtered/JKS002656_AnviProf/PROFILE.db -c JKS002656anvi.db

#JKS002656_Bins

anvi-script-reformat-fasta /home/egreen/MollicuteGenomes2021/Genomes_Meso_filtered/FiltJKS002657.fasta -o FiltJKS002657-fixed.fa -l 0 --simplify-names -r Keep2657tabs

anvi-gen-contigs-database -f FiltJKS002657-fixed.fa -o FiltJKS002657anvi.db -n anvitry2657

prokka --prefix PROKKA --outdir PROKKA_JKS002657-fixed --cpus 5 FiltJKS002657-fixed.fa --gcode 4 --kingdom Bacteria

python3 gff_parser.py PROKKA_JKS002657-fixed/PROKKA.gff --gene-calls FiltJKS002657gene_calls.txt --annotation FiltJKS002657gene_annot.txt

anvi-gen-contigs-database -f FiltJKS002657-fixed.fa -o FiltJKS002657anvi.db --external-gene-calls FiltJKS002657gene_calls.txt --ignore-internal-stop-codons -n anvitry2657

anvi-import-functions -c FiltJKS002657anvi.db -i FiltJKS002657gene_annot.txt

anvi-run-hmms -c FiltJKS002657anvi.db

anvi-display-contigs-stats FiltJKS002657anvi.db

#NEED TO MAKE BAM FILES WITH FIXED FASTA

bwa index ~/MollicuteGenomes2021/Genomes_Meso_filtered/FiltJKS002657-fixed.fa

bwa mem ~/MollicuteGenomes2021/Genomes_Meso_filtered/FiltJKS002657-fixed.fa ~/MollicuteGenomes2021/JKS002657_R1_paired.fastq ~/MollicuteGenomes2021/JKS002657_R2_paired.fastq > FiltJKS002657Aligned.sam -t 10

samtools index FiltJKS002657Aligned.sam

samtools fixmate -O bam FiltJKS002657Aligned.sam FiltJKS002657Aligned_fixmate.bam

samtools sort -o FiltJKS002657Aligned_sorted.bam -O bam -T temp FiltJKS002657Aligned_fixmate.bam

samtools index FiltJKS002657Aligned_sorted.bam

anvi-init-bam FiltJKS002657Aligned_sorted.bam -o FiltJKS002657.bam

anvi-run-scg-taxonomy -c FiltJKS002657anvi.db

anvi-profile -i FiltJKS002657.bam -c FiltJKS002657anvi.db --num-threads 10 --output-dir FiltJKS002657_AnviProf --min-contig-length 100 --sample-name FiltJKS002657 --cluster-contigs

anvi-interactive -p ~/MollicuteGenomes2021/Genomes_Meso_filtered/FiltJKS002657_AnviProf/PROFILE.db -c FiltJKS002657anvi.db

#FiltJKS002657_Bins

anvi-script-reformat-fasta /home/egreen/MollicuteGenomes2021/Genomes_Meso_filtered/FiltJKS002658.fasta -o FiltJKS002658-fixed.fa -l 0 --simplify-names -r Keep2658tabs

anvi-gen-contigs-database -f FiltJKS002658-fixed.fa -o FiltJKS002658anvi.db -n anvitry2658

prokka --prefix PROKKA --outdir PROKKA_JKS002658-fixed --cpus 5 FiltJKS002658-fixed.fa --gcode 4 --kingdom Bacteria

python3 gff_parser.py PROKKA_JKS002658-fixed/PROKKA.gff --gene-calls FiltJKS002658gene_calls.txt --annotation FiltJKS002658gene_annot.txt

anvi-gen-contigs-database -f FiltJKS002658-fixed.fa -o FiltJKS002658anvi.db --external-gene-calls FiltJKS002658gene_calls.txt --ignore-internal-stop-codons -n anvitry2658

anvi-import-functions -c FiltJKS002658anvi.db -i FiltJKS002658gene_annot.txt

anvi-run-hmms -c FiltJKS002658anvi.db

anvi-display-contigs-stats FiltJKS002658anvi.db

#NEED TO MAKE BAM FILES WITH FIXED FASTA

bwa index ~/MollicuteGenomes2021/Genomes_Meso_filtered/FiltJKS002658-fixed.fa

bwa mem ~/MollicuteGenomes2021/Genomes_Meso_filtered/FiltJKS002658-fixed.fa ~/MollicuteGenomes2021/JKS002658_R1_paired.fastq ~/MollicuteGenomes2021/JKS002658_R2_paired.fastq > FiltJKS002658Aligned.sam -t 10

samtools index FiltJKS002658Aligned.sam

samtools fixmate -O bam FiltJKS002658Aligned.sam FiltJKS002658Aligned_fixmate.bam

samtools sort -o FiltJKS002658Aligned_sorted.bam -O bam -T temp FiltJKS002658Aligned_fixmate.bam

samtools index FiltJKS002658Aligned_sorted.bam

anvi-init-bam FiltJKS002658Aligned_sorted.bam -o FiltJKS002658.bam

anvi-run-scg-taxonomy -c FiltJKS002658anvi.db

anvi-profile -i FiltJKS002658.bam -c FiltJKS002658anvi.db --num-threads 10 --output-dir FiltJKS002658_AnviProf --min-contig-length 100 --sample-name FiltJKS002658 --cluster-contigs

anvi-interactive -p ~/MollicuteGenomes2021/Genomes_Meso_filtered/FiltJKS002658_AnviProf/PROFILE.db -c FiltJKS002658anvi.db

#FiltJKS002658_Bins

anvi-script-reformat-fasta /home/egreen/MollicuteGenomes2021/Genomes_Meso_filtered/FiltJKS002659.fasta -o FiltJKS002659-fixed.fa -l 0 --simplify-names -r Keep2659tabs

anvi-gen-contigs-database -f FiltJKS002659-fixed.fa -o FiltJKS002659anvi.db -n anvitry2659

prokka --prefix PROKKA --outdir PROKKA_JKS002659-fixed --cpus 5 FiltJKS002659-fixed.fa --gcode 4 --kingdom Bacteria

python3 gff_parser.py PROKKA_JKS002659-fixed/PROKKA.gff --gene-calls FiltJKS002659gene_calls.txt --annotation FiltJKS002659gene_annot.txt

anvi-gen-contigs-database -f FiltJKS002659-fixed.fa -o FiltJKS002659anvi.db --external-gene-calls FiltJKS002659gene_calls.txt --ignore-internal-stop-codons -n anvitry2659

anvi-import-functions -c FiltJKS002659anvi.db -i FiltJKS002659gene_annot.txt

anvi-run-hmms -c FiltJKS002659anvi.db

anvi-display-contigs-stats FiltJKS002659anvi.db

#NEED TO MAKE BAM FILES WITH FIXED FASTA

bwa index ~/MollicuteGenomes2021/Genomes_Meso_filtered/FiltJKS002659-fixed.fa

bwa mem ~/MollicuteGenomes2021/Genomes_Meso_filtered/FiltJKS002659-fixed.fa ~/MollicuteGenomes2021/JKS002659_R1_paired.fastq ~/MollicuteGenomes2021/JKS002659_R2_paired.fastq > FiltJKS002659Aligned.sam -t 10

samtools index FiltJKS002659Aligned.sam

samtools fixmate -O bam FiltJKS002659Aligned.sam FiltJKS002659Aligned_fixmate.bam

samtools sort -o FiltJKS002659Aligned_sorted.bam -O bam -T temp FiltJKS002659Aligned_fixmate.bam

samtools index FiltJKS002659Aligned_sorted.bam

anvi-init-bam FiltJKS002659Aligned_sorted.bam -o FiltJKS002659.bam

anvi-run-scg-taxonomy -c FiltJKS002659anvi.db

anvi-profile -i FiltJKS002659.bam -c FiltJKS002659anvi.db --num-threads 10 --output-dir FiltJKS002659_AnviProf --min-contig-length 100 --sample-name FiltJKS002659 --cluster-contigs

anvi-interactive -p ~/MollicuteGenomes2021/Genomes_Meso_filtered/FiltJKS002659_AnviProf/PROFILE.db -c FiltJKS002659anvi.db

#FiltJKS002659_Bins

anvi-script-reformat-fasta /home/egreen/MollicuteGenomes2021/Genomes_Meso_filtered/FiltJKS002660.fasta -o FiltJKS002660-fixed.fa -l 0 --simplify-names -r Keep2660tabs

anvi-gen-contigs-database -f FiltJKS002660-fixed.fa -o FiltJKS002660anvi.db -n anvitry2660

prokka --prefix PROKKA --outdir PROKKA_JKS002660-fixed --cpus 5 FiltJKS002660-fixed.fa --gcode 4 --kingdom Bacteria

python3 gff_parser.py PROKKA_JKS002660-fixed/PROKKA.gff --gene-calls FiltJKS002660gene_calls.txt --annotation FiltJKS002660gene_annot.txt

anvi-gen-contigs-database -f FiltJKS002660-fixed.fa -o FiltJKS002660anvi.db --external-gene-calls FiltJKS002660gene_calls.txt --ignore-internal-stop-codons -n anvitry2660

anvi-import-functions -c FiltJKS002660anvi.db -i FiltJKS002660gene_annot.txt

anvi-run-hmms -c FiltJKS002660anvi.db

anvi-display-contigs-stats FiltJKS002660anvi.db

#NEED TO MAKE BAM FILES WITH FIXED FASTA

bwa index ~/MollicuteGenomes2021/Genomes_Meso_filtered/FiltJKS002660-fixed.fa

bwa mem ~/MollicuteGenomes2021/Genomes_Meso_filtered/FiltJKS002660-fixed.fa ~/MollicuteGenomes2021/ReRanLibraryCheck/JKS002660_R1_paired.fastq ~/MollicuteGenomes2021/ReRanLibraryCheck/JKS002660_R2_paired.fastq > FiltJKS002660Aligned.sam -t 10

samtools index FiltJKS002660Aligned.sam

samtools fixmate -O bam FiltJKS002660Aligned.sam FiltJKS002660Aligned_fixmate.bam

samtools sort -o FiltJKS002660Aligned_sorted.bam -O bam -T temp FiltJKS002660Aligned_fixmate.bam

samtools index FiltJKS002660Aligned_sorted.bam

anvi-init-bam FiltJKS002660Aligned_sorted.bam -o FiltJKS002660.bam

anvi-run-scg-taxonomy -c FiltJKS002660anvi.db

anvi-profile -i FiltJKS002660.bam -c FiltJKS002660anvi.db --num-threads 10 --output-dir FiltJKS002660_AnviProf --min-contig-length 100 --sample-name FiltJKS002660 --cluster-contigs

anvi-interactive -p ~/MollicuteGenomes2021/Genomes_Meso_filtered/FiltJKS002660_AnviProf/PROFILE.db -c FiltJKS002660anvi.db

#FiltJKS002660_Bins

anvi-script-reformat-fasta /home/egreen/MollicuteGenomes2021/Genomes_Meso_filtered/FiltJKS002661.fasta -o FiltJKS002661-fixed.fa -l 0 --simplify-names -r Keep2661tabs

anvi-gen-contigs-database -f FiltJKS002661-fixed.fa -o FiltJKS002661anvi.db -n anvitry2661

prokka --prefix PROKKA --outdir PROKKA_JKS002661-fixed --cpus 5 FiltJKS002661-fixed.fa --gcode 4 --kingdom Bacteria

python3 gff_parser.py PROKKA_JKS002661-fixed/PROKKA.gff --gene-calls FiltJKS002661gene_calls.txt --annotation FiltJKS002661gene_annot.txt

anvi-gen-contigs-database -f FiltJKS002661-fixed.fa -o FiltJKS002661anvi.db --external-gene-calls FiltJKS002661gene_calls.txt --ignore-internal-stop-codons -n anvitry2661

anvi-import-functions -c FiltJKS002661anvi.db -i FiltJKS002661gene_annot.txt

anvi-run-hmms -c FiltJKS002661anvi.db

anvi-display-contigs-stats FiltJKS002661anvi.db

#NEED TO MAKE BAM FILES WITH FIXED FASTA

bwa index ~/MollicuteGenomes2021/Genomes_Meso_filtered/FiltJKS002661-fixed.fa

bwa mem ~/MollicuteGenomes2021/Genomes_Meso_filtered/FiltJKS002661-fixed.fa ~/MollicuteGenomes2021/ReRanLibraryCheck/JKS002661_R1_paired.fastq ~/MollicuteGenomes2021/ReRanLibraryCheck/JKS002661_R2_paired.fastq > FiltJKS002661Aligned.sam -t 10

samtools index FiltJKS002661Aligned.sam

samtools fixmate -O bam FiltJKS002661Aligned.sam FiltJKS002661Aligned_fixmate.bam

samtools sort -o FiltJKS002661Aligned_sorted.bam -O bam -T temp FiltJKS002661Aligned_fixmate.bam

samtools index FiltJKS002661Aligned_sorted.bam

anvi-init-bam FiltJKS002661Aligned_sorted.bam -o FiltJKS002661.bam

anvi-run-scg-taxonomy -c FiltJKS002661anvi.db

anvi-profile -i FiltJKS002661.bam -c FiltJKS002661anvi.db --num-threads 10 --output-dir FiltJKS002661_AnviProf --min-contig-length 100 --sample-name FiltJKS002661 --cluster-contigs

anvi-interactive -p ~/MollicuteGenomes2021/Genomes_Meso_filtered/FiltJKS002661_AnviProf/PROFILE.db -c FiltJKS002661anvi.db

#FiltJKS002661_Bins

anvi-script-reformat-fasta /home/egreen/MollicuteGenomes2021/Genomes_Meso_filtered/FiltJKS002662.fasta -o FiltJKS002662-fixed.fa -l 0 --simplify-names -r Keep2662tabs

anvi-gen-contigs-database -f FiltJKS002662-fixed.fa -o FiltJKS002662anvi.db -n anvitry2662

prokka --prefix PROKKA --outdir PROKKA_JKS002662-fixed --cpus 5 FiltJKS002662-fixed.fa --gcode 4 --kingdom Bacteria

python3 gff_parser.py PROKKA_JKS002662-fixed/PROKKA.gff --gene-calls FiltJKS002662gene_calls.txt --annotation FiltJKS002662gene_annot.txt

anvi-gen-contigs-database -f FiltJKS002662-fixed.fa -o FiltJKS002662anvi.db --external-gene-calls FiltJKS002662gene_calls.txt --ignore-internal-stop-codons -n anvitry2662

anvi-import-functions -c FiltJKS002662anvi.db -i FiltJKS002662gene_annot.txt

anvi-run-hmms -c FiltJKS002662anvi.db

anvi-display-contigs-stats FiltJKS002662anvi.db

#NEED TO MAKE BAM FILES WITH FIXED FASTA

bwa index ~/MollicuteGenomes2021/Genomes_Meso_filtered/FiltJKS002662-fixed.fa

bwa mem ~/MollicuteGenomes2021/Genomes_Meso_filtered/FiltJKS002662-fixed.fa ~/MollicuteGenomes2021/ReRanLibraryCheck/JKS002662_R1_paired.fastq ~/MollicuteGenomes2021/ReRanLibraryCheck/JKS002662_R2_paired.fastq > FiltJKS002662Aligned.sam -t 10

samtools index FiltJKS002662Aligned.sam

samtools fixmate -O bam FiltJKS002662Aligned.sam FiltJKS002662Aligned_fixmate.bam

samtools sort -o FiltJKS002662Aligned_sorted.bam -O bam -T temp FiltJKS002662Aligned_fixmate.bam

samtools index FiltJKS002662Aligned_sorted.bam

anvi-init-bam FiltJKS002662Aligned_sorted.bam -o FiltJKS002662.bam

anvi-run-scg-taxonomy -c FiltJKS002662anvi.db

anvi-profile -i FiltJKS002662.bam -c FiltJKS002662anvi.db --num-threads 10 --output-dir FiltJKS002662_AnviProf --min-contig-length 100 --sample-name FiltJKS002662 --cluster-contigs

anvi-interactive -p ~/MollicuteGenomes2021/Genomes_Meso_filtered/FiltJKS002662_AnviProf/PROFILE.db -c FiltJKS002662anvi.db

#FiltJKS002662_Bins

anvi-script-reformat-fasta /home/egreen/MollicuteGenomes2021/Genomes_Meso_filtered/FiltJKS002663.fasta -o FiltJKS002663-fixed.fa -l 0 --simplify-names -r Keep2663tabs

anvi-gen-contigs-database -f FiltJKS002663-fixed.fa -o FiltJKS002663anvi.db -n anvitry2663

prokka --prefix PROKKA --outdir PROKKA_JKS002663-fixed --cpus 5 FiltJKS002663-fixed.fa --gcode 4 --kingdom Bacteria

python3 gff_parser.py PROKKA_JKS002663-fixed/PROKKA.gff --gene-calls FiltJKS002663gene_calls.txt --annotation FiltJKS002663gene_annot.txt

anvi-gen-contigs-database -f FiltJKS002663-fixed.fa -o FiltJKS002663anvi.db --external-gene-calls FiltJKS002663gene_calls.txt --ignore-internal-stop-codons -n anvitry2663

anvi-import-functions -c FiltJKS002663anvi.db -i FiltJKS002663gene_annot.txt

anvi-run-hmms -c FiltJKS002663anvi.db

anvi-display-contigs-stats FiltJKS002663anvi.db

#NEED TO MAKE BAM FILES WITH FIXED FAST

bwa index ~/MollicuteGenomes2021/Genomes_Meso_filtered/FiltJKS002663-fixed.fa

bwa mem ~/MollicuteGenomes2021/Genomes_Meso_filtered/FiltJKS002663-fixed.fa ~/MollicuteGenomes2021/ReRanLibraryCheck/JKS002663_R1_paired.fastq ~/MollicuteGenomes2021/ReRanLibraryCheck/JKS002663_R2_paired.fastq > FiltJKS002663Aligned.sam -t 10

samtools index FiltJKS002663Aligned.sam

samtools fixmate -O bam FiltJKS002663Aligned.sam FiltJKS002663Aligned_fixmate.bam

samtools sort -o FiltJKS002663Aligned_sorted.bam -O bam -T temp FiltJKS002663Aligned_fixmate.bam

samtools index FiltJKS002663Aligned_sorted.bam

anvi-init-bam FiltJKS002663Aligned_sorted.bam -o FiltJKS002663.bam

anvi-run-scg-taxonomy -c FiltJKS002663anvi.db

anvi-profile -i FiltJKS002663.bam -c FiltJKS002663anvi.db --num-threads 10 --output-dir FiltJKS002663_AnviProf --min-contig-length 100 --sample-name FiltJKS002663 --cluster-contigs

anvi-interactive -p ~/MollicuteGenomes2021/Genomes_Meso_filtered/FiltJKS002663_AnviProf/PROFILE.db -c FiltJKS002663anvi.db

#FiltJKS002663_Bins

anvi-script-reformat-fasta /home/egreen/MollicuteGenomes2021/Genomes_Meso_filtered/FiltJKS002664.fasta -o FiltJKS002664-fixed.fa -l 0 --simplify-names -r Keep2664tabs

anvi-gen-contigs-database -f FiltJKS002664-fixed.fa -o FiltJKS002664anvi.db -n anvitry2664

prokka --prefix PROKKA --outdir PROKKA_JKS002664-fixed --cpus 5 FiltJKS002664-fixed.fa --gcode 4 --kingdom Bacteria

python3 gff_parser.py PROKKA_JKS002664-fixed/PROKKA.gff --gene-calls FiltJKS002664gene_calls.txt --annotation FiltJKS002664gene_annot.txt

anvi-gen-contigs-database -f FiltJKS002664-fixed.fa -o FiltJKS002664anvi.db --external-gene-calls FiltJKS002664gene_calls.txt --ignore-internal-stop-codons -n anvitry2664

anvi-import-functions -c FiltJKS002664anvi.db -i FiltJKS002664gene_annot.txt

anvi-run-hmms -c FiltJKS002664anvi.db

anvi-display-contigs-stats FiltJKS002664anvi.db

#NEED TO MAKE BAM FILES WITH FIXED FAST

bwa index ~/MollicuteGenomes2021/Genomes_Meso_filtered/FiltJKS002664-fixed.fa

bwa mem ~/MollicuteGenomes2021/Genomes_Meso_filtered/FiltJKS002664-fixed.fa ~/MollicuteGenomes2021/ReRanLibraryCheck/JKS002664_R1_paired.fastq ~/MollicuteGenomes2021/ReRanLibraryCheck/JKS002664_R2_paired.fastq > FiltJKS002664Aligned.sam -t 10

samtools index FiltJKS002664Aligned.sam

samtools fixmate -O bam FiltJKS002664Aligned.sam FiltJKS002664Aligned_fixmate.bam

samtools sort -o FiltJKS002664Aligned_sorted.bam -O bam -T temp FiltJKS002664Aligned_fixmate.bam

samtools index FiltJKS002664Aligned_sorted.bam

anvi-init-bam FiltJKS002664Aligned_sorted.bam -o FiltJKS002664.bam

anvi-run-scg-taxonomy -c FiltJKS002664anvi.db

anvi-profile -i FiltJKS002664.bam -c FiltJKS002664anvi.db --num-threads 10 --output-dir FiltJKS002664_AnviProf --min-contig-length 100 --sample-name FiltJKS002664 --cluster-contigs

anvi-interactive -p ~/MollicuteGenomes2021/Genomes_Meso_filtered/FiltJKS002664_AnviProf/PROFILE.db -c FiltJKS002664anvi.db

#FiltJKS002664_Bins

#JGIMeta files

#TSFL002

anvi-script-reformat-fasta ~/MollicuteGenomes2021/JGIMeta/TSFL002_MetaG.fasta -o TSFL002-fixed.fa -l 0 --simplify-names -r KeepTSFL002tabs

anvi-gen-contigs-database -f TSFL002-fixed.fa -o TSFL002anvi.db -n anvitryTSFL002

prokka --prefix PROKKA --outdir PROKKA_TSFL002-fixed --cpus 5 TSFL002-fixed.fa --gcode 4 --kingdom Bacteria

python3 gff_parser.py PROKKA_TSFL002-fixed/PROKKA.gff --gene-calls TSFL002gene_calls.txt --annotation TSFL002gene_annot.txt

anvi-gen-contigs-database -f TSFL002-fixed.fa -o TSFL002anvi.db --external-gene-calls TSFL002gene_calls.txt --ignore-internal-stop-codons -n anvitryTSFL002

anvi-import-functions -c TSFL002anvi.db -i TSFL002gene_annot.txt

anvi-run-hmms -c TSFL002anvi.db

anvi-display-contigs-stats TSFL002anvi.db

#NEED TO MAKE BAM FILES WITH FIXED FASTA

bwa index TSFL002-fixed.fa

bwa mem TSFL002-fixed.fa 10975.3.186416.ATAGCGG-ACCGCTA.filter-METAGENOME.fastq > TSFL002Aligned.sam -t 10

samtools index TSFL002Aligned.sam

samtools fixmate -O bam TSFL002Aligned.sam TSFL002Aligned_fixmate.bam

samtools sort -o TSFL002Aligned_sorted.bam -O bam -T temp TSFL002Aligned_fixmate.bam

samtools index TSFL002Aligned_sorted.bam

anvi-init-bam TSFL002Aligned_sorted.bam -o TSFL002.bam

anvi-profile -i TSFL002.bam -c TSFL002anvi.db --num-threads 10 --output-dir TSFL002_AnviProf --min-contig-length 100 --sample-name TSFL002 --cluster-contigs

anvi-run-scg-taxonomy -c TSFL002anvi.db

anvi-interactive -p ~/MollicuteGenomes2021/JGIMeta/TSFL002_AnviProf/PROFILE.db -c TSFL002anvi.db

#TSFL002_Bins

#TSFL042

anvi-script-reformat-fasta ~/MollicuteGenomes2021/JGIMeta/TSFL042_MetaG.fasta -o TSFL042-fixed.fa -l 0 --simplify-names -r KeepTSFL042tabs

anvi-gen-contigs-database -f TSFL042-fixed.fa -o TSFL042anvi.db -n anvitryTSFL042 --num-threads 10

prokka --prefix PROKKA --outdir PROKKA_TSFL042-fixed --cpus 5 TSFL042-fixed.fa --gcode 4 --kingdom Bacteria

python3 gff_parser.py PROKKA_TSFL042-fixed/PROKKA.gff --gene-calls TSFL042gene_calls.txt --annotation TSFL042gene_annot.txt

anvi-gen-contigs-database -f TSFL042-fixed.fa -o TSFL042anvi.db --external-gene-calls TSFL042gene_calls.txt --ignore-internal-stop-codons -n anvitryTSFL042

anvi-import-functions -c TSFL042anvi.db -i TSFL042gene_annot.txt

anvi-run-hmms -c TSFL042anvi.db --num-threads 10

#anvi-display-contigs-stats TSFL042anvi.db

#NEED TO MAKE BAM FILES WITH FIXED FASTA

bwa index TSFL042-fixed.fa

bwa mem TSFL042-fixed.fa 10975.6.186451.AGCAAGC-TGCTTGC.filter-TSFL042.fastq > TSFL042Aligned.sam -t 10

samtools index TSFL042Aligned.sam

samtools fixmate -O bam TSFL042Aligned.sam TSFL042Aligned_fixmate.bam

samtools sort -o TSFL042Aligned_sorted.bam -O bam -T temp TSFL042Aligned_fixmate.bam

samtools index TSFL042Aligned_sorted.bam

anvi-init-bam TSFL042Aligned_sorted.bam -o TSFL042.bam --num-threads 10

anvi-profile -i TSFL042.bam -c TSFL042anvi.db --num-threads 10 --output-dir TSFL042_AnviProf --min-contig-length 100 --sample-name TSFL042 --cluster-contigs

anvi-run-scg-taxonomy -c TSFL042anvi.db

anvi-interactive -p ~/MollicuteGenomes2021/JGIMeta/TSFL042_AnviProf/PROFILE.db -c TSFL042anvi.db

#TSFL042_Bins

#TSFL050

anvi-script-reformat-fasta ~/MollicuteGenomes2021/JGIMeta/TSFL050_MetaG.fasta -o TSFL050-fixed.fa -l 0 --simplify-names -r KeepTSFL050tabs

anvi-gen-contigs-database -f TSFL050-fixed.fa -o TSFL050anvi.db -n anvitryTSFL050 --num-threads 10

prokka --prefix PROKKA --outdir PROKKA_TSFL050-fixed --cpus 5 TSFL050-fixed.fa --gcode 4 --kingdom Bacteria

python3 gff_parser.py PROKKA_TSFL050-fixed/PROKKA.gff --gene-calls TSFL050gene_calls.txt --annotation TSFL050gene_annot.txt

anvi-gen-contigs-database -f TSFL050-fixed.fa -o TSFL050anvi.db --external-gene-calls TSFL050gene_calls.txt --ignore-internal-stop-codons -n anvitryTSFL050

anvi-import-functions -c TSFL050anvi.db -i TSFL050gene_annot.txt

anvi-run-hmms -c TSFL050anvi.db --num-threads 10

#anvi-display-contigs-stats TSFL050anvi.db

#NEED TO MAKE BAM FILES WITH FIXED FASTA

bwa index TSFL050-fixed.fa

bwa mem TSFL050-fixed.fa 10975.5.186440.TGGATCA-GTGATCC.filter-TSFL050.fastq > TSFL050Aligned.sam -t 15

samtools index TSFL050Aligned.sam

samtools fixmate -O bam TSFL050Aligned.sam TSFL050Aligned_fixmate.bam

samtools sort -o TSFL050Aligned_sorted.bam -O bam -T temp TSFL050Aligned_fixmate.bam

samtools index TSFL050Aligned_sorted.bam

anvi-init-bam TSFL050Aligned_sorted.bam -o TSFL050.bam --num-threads 10

anvi-profile -i TSFL050.bam -c TSFL050anvi.db --num-threads 10 --output-dir TSFL050_AnviProf --min-contig-length 100 --sample-name TSFL050 --cluster-contigs

anvi-run-scg-taxonomy -c TSFL050anvi.db

anvi-interactive -p ~/MollicuteGenomes2021/JGIMeta/TSFL050_AnviProf/PROFILE.db -c TSFL050anvi.db

#TSFL050_Bins

#TSNJ022

anvi-script-reformat-fasta ~/MollicuteGenomes2021/JGIMeta/TSNJ022_MetaG.fasta -o TSNJ022-fixed.fa -l 0 --simplify-names -r KeepTSNJ022tabs

anvi-gen-contigs-database -f TSNJ022-fixed.fa -o TSNJ022anvi.db -n anvitryTSNJ022 --num-threads 10

prokka --prefix PROKKA --outdir PROKKA_TSNJ022-fixed --cpus 5 TSNJ022-fixed.fa --gcode 4 --kingdom Bacteria

python3 gff_parser.py PROKKA_TSNJ022-fixed/PROKKA.gff --gene-calls TSNJ022gene_calls.txt --annotation TSNJ022gene_annot.txt

anvi-gen-contigs-database -f TSNJ022-fixed.fa -o TSNJ022anvi.db --external-gene-calls TSNJ022gene_calls.txt --ignore-internal-stop-codons -n anvitryTSNJ022

anvi-import-functions -c TSNJ022anvi.db -i TSNJ022gene_annot.txt

anvi-run-hmms -c TSNJ022anvi.db --num-threads 10

#anvi-display-contigs-stats TSNJ022anvi.db

#NEED TO MAKE BAM FILES WITH FIXED FASTA

bwa index TSNJ022-fixed.fa

bwa mem TSNJ022-fixed.fa 10976.1.186488.AGTCTCA-GTGAGAC.filter-TSNJ022.fastq > TSNJ022Aligned.sam -t 10

samtools index TSNJ022Aligned.sam

samtools fixmate -O bam TSNJ022Aligned.sam TSNJ022Aligned_fixmate.bam

samtools sort -o TSNJ022Aligned_sorted.bam -O bam -T temp TSNJ022Aligned_fixmate.bam

samtools index TSNJ022Aligned_sorted.bam

anvi-init-bam TSNJ022Aligned_sorted.bam -o TSNJ022.bam --num-threads 10

anvi-profile -i TSNJ022.bam -c TSNJ022anvi.db --num-threads 10 --output-dir TSNJ022_AnviProf --min-contig-length 100 --sample-name TSNJ022 --cluster-contigs

anvi-run-scg-taxonomy -c TSNJ022anvi.db

anvi-interactive -p ~/MollicuteGenomes2021/JGIMeta/TSNJ022_AnviProf/PROFILE.db -c TSNJ022anvi.db

# "TSNJ022_Bins"

#TSNJ026

anvi-script-reformat-fasta ~/MollicuteGenomes2021/JGIMeta/TSNJ026_MetaG.fasta -o TSNJ026-fixed.fa -l 0 --simplify-names -r KeepTSNJ026tabs

anvi-gen-contigs-database -f TSNJ026-fixed.fa -o TSNJ026anvi.db -n anvitryTSNJ026 --num-threads 10

prokka --prefix PROKKA --outdir PROKKA_TSNJ026-fixed --cpus 5 TSNJ026-fixed.fa --gcode 4 --kingdom Bacteria

python3 gff_parser.py PROKKA_TSNJ026-fixed/PROKKA.gff --gene-calls TSNJ026gene_calls.txt --annotation TSNJ026gene_annot.txt

anvi-gen-contigs-database -f TSNJ026-fixed.fa -o TSNJ026anvi.db --external-gene-calls TSNJ026gene_calls.txt --ignore-internal-stop-codons -n anvitryTSNJ026

anvi-import-functions -c TSNJ026anvi.db -i TSNJ026gene_annot.txt

anvi-run-hmms -c TSNJ026anvi.db --num-threads 10

#anvi-display-contigs-stats TSNJ026anvi.db

#NEED TO MAKE BAM FILES WITH FIXED FASTA

bwa index TSNJ026-fixed.fa

bwa mem TSNJ026-fixed.fa 10975.5.186440.GTAACGA-GTCGTTA.filter-TSNJ026.fastq > TSNJ026Aligned.sam -t 10

samtools index TSNJ026Aligned.sam

samtools fixmate -O bam TSNJ026Aligned.sam TSNJ026Aligned_fixmate.bam

samtools sort -o TSNJ026Aligned_sorted.bam -O bam -T temp TSNJ026Aligned_fixmate.bam

samtools index TSNJ026Aligned_sorted.bam

anvi-init-bam TSNJ026Aligned_sorted.bam -o TSNJ026.bam --num-threads 10

anvi-profile -i TSNJ026.bam -c TSNJ026anvi.db --num-threads 10 --output-dir TSNJ026_AnviProf --min-contig-length 100 --sample-name TSNJ026 --cluster-contigs

anvi-run-scg-taxonomy -c TSNJ026anvi.db

anvi-interactive -p ~/MollicuteGenomes2021/JGIMeta/TSNJ026_AnviProf/PROFILE.db -c TSNJ026anvi.db

#TSNJ026_Bins

#TSNJ034

anvi-script-reformat-fasta ~/MollicuteGenomes2021/JGIMeta/TSNJ034_MetaG.fasta -o TSNJ034-fixed.fa -l 0 --simplify-names -r KeepTSNJ034tabs

anvi-gen-contigs-database -f TSNJ034-fixed.fa -o TSNJ034anvi.db -n anvitryTSNJ034 --num-threads 10

prokka --prefix PROKKA --outdir PROKKA_TSNJ034-fixed --cpus 5 TSNJ034-fixed.fa --gcode 4 --kingdom Bacteria

python3 gff_parser.py PROKKA_TSNJ034-fixed/PROKKA.gff --gene-calls TSNJ034gene_calls.txt --annotation TSNJ034gene_annot.txt

anvi-gen-contigs-database -f TSNJ034-fixed.fa -o TSNJ034anvi.db --external-gene-calls TSNJ034gene_calls.txt --ignore-internal-stop-codons -n anvitryTSNJ034

anvi-import-functions -c TSNJ034anvi.db -i TSNJ034gene_annot.txt

anvi-run-hmms -c TSNJ034anvi.db --num-threads 10

#anvi-display-contigs-stats TSNJ034anvi.db

#NEED TO MAKE BAM FILES WITH FIXED FASTA

bwa index TSNJ034-fixed.fa

bwa mem TSNJ034-fixed.fa 10975.5.186440.ACGATGA-GTCATCG.filter-TJNJ034.fastq > TSNJ034Aligned.sam -t 10

samtools index TSNJ034Aligned.sam

samtools fixmate -O bam TSNJ034Aligned.sam TSNJ034Aligned_fixmate.bam

samtools sort -o TSNJ034Aligned_sorted.bam -O bam -T temp TSNJ034Aligned_fixmate.bam

samtools index TSNJ034Aligned_sorted.bam

anvi-init-bam TSNJ034Aligned_sorted.bam -o TSNJ034.bam --num-threads 10

anvi-profile -i TSNJ034.bam -c TSNJ034anvi.db --num-threads 10 --output-dir TSNJ034_AnviProf --min-contig-length 100 --sample-name TSNJ034 --cluster-contigs

anvi-run-scg-taxonomy -c TSNJ034anvi.db

anvi-interactive -p ~/MollicuteGenomes2021/JGIMeta/TSNJ034_AnviProf/PROFILE.db -c TSNJ034anvi.db

#TSNJ034_Bins

#TSNJ035

anvi-script-reformat-fasta ~/MollicuteGenomes2021/JGIMeta/TSNJ035_MetaG.fasta -o TSNJ035-fixed.fa -l 0 --simplify-names -r KeepTSNJ035tabs

anvi-gen-contigs-database -f TSNJ035-fixed.fa -o TSNJ035anvi.db -n anvitryTSNJ035 --num-threads 10

prokka --prefix PROKKA --outdir PROKKA_TSNJ035-fixed --cpus 5 TSNJ035-fixed.fa --gcode 4 --kingdom Bacteria

python3 gff_parser.py PROKKA_TSNJ035-fixed/PROKKA.gff --gene-calls TSNJ035gene_calls.txt --annotation TSNJ035gene_annot.txt

anvi-gen-contigs-database -f TSNJ035-fixed.fa -o TSNJ035anvi.db --external-gene-calls TSNJ035gene_calls.txt --ignore-internal-stop-codons -n anvitryTSNJ035

anvi-import-functions -c TSNJ035anvi.db -i TSNJ035gene_annot.txt

anvi-run-hmms -c TSNJ035anvi.db --num-threads 10

#anvi-display-contigs-stats TSNJ035anvi.db

#NEED TO MAKE BAM FILES WITH FIXED FASTA

bwa index TSNJ035-fixed.fa

bwa mem TSNJ035-fixed.fa 10976.2.186501.TACGCCT-AAGGCGT.filter-TSNJ035.fastq > TSNJ035Aligned.sam -t 10

samtools index TSNJ035Aligned.sam

samtools fixmate -O bam TSNJ035Aligned.sam TSNJ035Aligned_fixmate.bam

samtools sort -o TSNJ035Aligned_sorted.bam -O bam -T temp TSNJ035Aligned_fixmate.bam

samtools index TSNJ035Aligned_sorted.bam

anvi-init-bam TSNJ035Aligned_sorted.bam -o TSNJ035.bam --num-threads 10

anvi-profile -i TSNJ035.bam -c TSNJ035anvi.db --num-threads 10 --output-dir TSNJ035_AnviProf --min-contig-length 100 --sample-name TSNJ035 --cluster-contigs

anvi-run-scg-taxonomy -c TSNJ035anvi.db

anvi-interactive -p ~/MollicuteGenomes2021/JGIMeta/TSNJ035_AnviProf/PROFILE.db -c TSNJ035anvi.db

#TSNJ035_Bins

#TSNJ092

anvi-script-reformat-fasta ~/MollicuteGenomes2021/JGIMeta/TSNJ092_MetaG.fasta -o TSNJ092-fixed.fa -l 0 --simplify-names -r KeepTSNJ092tabs

anvi-gen-contigs-database -f TSNJ092-fixed.fa -o TSNJ092anvi.db -n anvitryTSNJ092 --num-threads 10

prokka --prefix PROKKA --outdir PROKKA_TSNJ092-fixed --cpus 5 TSNJ092-fixed.fa --gcode 4 --kingdom Bacteria

python3 gff_parser.py PROKKA_TSNJ092-fixed/PROKKA.gff --gene-calls TSNJ092gene_calls.txt --annotation TSNJ092gene_annot.txt

anvi-gen-contigs-database -f TSNJ092-fixed.fa -o TSNJ092anvi.db --external-gene-calls TSNJ092gene_calls.txt --ignore-internal-stop-codons -n anvitryTSNJ092

anvi-import-functions -c TSNJ092anvi.db -i TSNJ092gene_annot.txt

anvi-run-hmms -c TSNJ092anvi.db --num-threads 10

#anvi-display-contigs-stats TSNJ092anvi.db

#NEED TO MAKE BAM FILES WITH FIXED FASTA

bwa index TSNJ092-fixed.fa

bwa mem TSNJ092-fixed.fa 10975.8.186475.AGCTCCT-TAGGAGC.filter-TSNJ092.fastq > TSNJ092Aligned.sam -t 10

samtools index TSNJ092Aligned.sam

samtools fixmate -O bam TSNJ092Aligned.sam TSNJ092Aligned_fixmate.bam

samtools sort -o TSNJ092Aligned_sorted.bam -O bam -T temp TSNJ092Aligned_fixmate.bam

samtools index TSNJ092Aligned_sorted.bam

anvi-init-bam TSNJ092Aligned_sorted.bam -o TSNJ092.bam --num-threads 10

anvi-profile -i TSNJ092.bam -c TSNJ092anvi.db --num-threads 10 --output-dir TSNJ092_AnviProf --min-contig-length 100 --sample-name TSNJ092 --cluster-contigs

anvi-run-scg-taxonomy -c TSNJ092anvi.db

anvi-interactive -p ~/MollicuteGenomes2021/JGIMeta/TSNJ092_AnviProf/PROFILE.db -c TSNJ092anvi.db

#TSNJ092_Bins

#TSNC071

anvi-script-reformat-fasta ~/MollicuteGenomes2021/JGIMeta/TSNC071_MetaG.fasta -o TSNC071-fixed.fa -l 0 --simplify-names -r KeepTSNC071tabs

anvi-gen-contigs-database -f TSNC071-fixed.fa -o TSNC071anvi.db -n anvitryTSNC071 --num-threads 10

prokka --prefix PROKKA --outdir PROKKA_TSNC071-fixed --cpus 5 TSNC071-fixed.fa --gcode 4 --kingdom Bacteria

python3 gff_parser.py PROKKA_TSNC071-fixed/PROKKA.gff --gene-calls TSNC071gene_calls.txt --annotation TSNC071gene_annot.txt

anvi-gen-contigs-database -f TSNC071-fixed.fa -o TSNC071anvi.db --external-gene-calls TSNC071gene_calls.txt --ignore-internal-stop-codons -n anvitryTSNC071

anvi-import-functions -c TSNC071anvi.db -i TSNC071gene_annot.txt

anvi-run-hmms -c TSNC071anvi.db --num-threads 10

#anvi-display-contigs-stats TSNC071anvi.db

#NEED TO MAKE BAM FILES WITH FIXED FASTA

bwa index TSNC071-fixed.fa

bwa mem TSNC071-fixed.fa 10976.2.186501.CCATACG-ACGTATG.filter-TSNC071.fastq > TSNC071Aligned.sam -t 10

samtools index TSNC071Aligned.sam

samtools fixmate -O bam TSNC071Aligned.sam TSNC071Aligned_fixmate.bam

samtools sort -o TSNC071Aligned_sorted.bam -O bam -T temp TSNC071Aligned_fixmate.bam

samtools index TSNC071Aligned_sorted.bam

anvi-init-bam TSNC071Aligned_sorted.bam -o TSNC071.bam --num-threads 10

anvi-profile -i TSNC071.bam -c TSNC071anvi.db --num-threads 10 --output-dir TSNC071_AnviProf --min-contig-length 100 --sample-name TSNC071 --cluster-contigs

anvi-run-scg-taxonomy -c TSNC071anvi.db

anvi-interactive -p ~/MollicuteGenomes2021/JGIMeta/TSNC071_AnviProf/PROFILE.db -c TSNC071anvi.db

#TSNC071_Bins

#TSGA003

anvi-script-reformat-fasta ~/MollicuteGenomes2021/JGIMeta/TSGA003_MetaG.fasta -o TSGA003-fixed.fa -l 0 --simplify-names -r KeepTSGA003tabs

anvi-gen-contigs-database -f TSGA003-fixed.fa -o TSGA003anvi.db -n anvitryTSGA003 --num-threads 10

prokka --prefix PROKKA --outdir PROKKA_TSGA003-fixed --cpus 5 TSGA003-fixed.fa --gcode 4 --kingdom Bacteria

python3 gff_parser.py PROKKA_TSGA003-fixed/PROKKA.gff --gene-calls TSGA003gene_calls.txt --annotation TSGA003gene_annot.txt

anvi-gen-contigs-database -f TSGA003-fixed.fa -o TSGA003anvi.db --external-gene-calls TSGA003gene_calls.txt --ignore-internal-stop-codons -n anvitryTSGA003

anvi-import-functions -c TSGA003anvi.db -i TSGA003gene_annot.txt

anvi-run-hmms -c TSGA003anvi.db --num-threads 10

#anvi-display-contigs-stats TSGA003anvi.db

#NEED TO MAKE BAM FILES WITH FIXED FASTA

bwa index TSGA003-fixed.fa

bwa mem TSGA003-fixed.fa 10975.8.186475.CGGTTGT-AACAACC.filterTSGA003.fastq > TSGA003Aligned.sam -t 10

samtools index TSGA003Aligned.sam

samtools fixmate -O bam TSGA003Aligned.sam TSGA003Aligned_fixmate.bam

samtools sort -o TSGA003Aligned_sorted.bam -O bam -T temp TSGA003Aligned_fixmate.bam

samtools index TSGA003Aligned_sorted.bam

anvi-init-bam TSGA003Aligned_sorted.bam -o TSGA003.bam --num-threads 10

anvi-profile -i TSGA003.bam -c TSGA003anvi.db --num-threads 10 --output-dir TSGA003_AnviProf --min-contig-length 100 --sample-name TSGA003 --cluster-contigs

anvi-run-scg-taxonomy -c TSGA003anvi.db

anvi-interactive -p ~/MollicuteGenomes2021/JGIMeta/TSGA003_AnviProf/PROFILE.db -c TSGA003anvi.db

#TSGA003_Bins

#TSGA061

anvi-script-reformat-fasta ~/MollicuteGenomes2021/JGIMeta/TSGA061_MetaG.fasta -o TSGA061-fixed.fa -l 0 --simplify-names -r KeepTSGA061tabs

anvi-gen-contigs-database -f TSGA061-fixed.fa -o TSGA061anvi.db -n anvitryTSGA061 --num-threads 10

prokka --prefix PROKKA --outdir PROKKA_TSGA061-fixed --cpus 5 TSGA061-fixed.fa --gcode 4 --kingdom Bacteria

python3 gff_parser.py PROKKA_TSGA061-fixed/PROKKA.gff --gene-calls TSGA061gene_calls.txt --annotation TSGA061gene_annot.txt

anvi-gen-contigs-database -f TSGA061-fixed.fa -o TSGA061anvi.db --external-gene-calls TSGA061gene_calls.txt --ignore-internal-stop-codons -n anvitryTSGA061

anvi-import-functions -c TSGA061anvi.db -i TSGA061gene_annot.txt

anvi-run-hmms -c TSGA061anvi.db --num-threads 10

#anvi-display-contigs-stats TSGA061anvi.db

#NEED TO MAKE BAM FILES WITH FIXED FASTA

bwa index TSGA061-fixed.fa

bwa mem TSGA061-fixed.fa 10975.8.186475.TTGTCGG-ACCGACA.filter-TSGA061.fastq > TSGA061Aligned.sam -t 10

samtools index TSGA061Aligned.sam

samtools fixmate -O bam TSGA061Aligned.sam TSGA061Aligned_fixmate.bam

samtools sort -o TSGA061Aligned_sorted.bam -O bam -T temp TSGA061Aligned_fixmate.bam

samtools index TSGA061Aligned_sorted.bam

anvi-init-bam TSGA061Aligned_sorted.bam -o TSGA061.bam --num-threads 10

anvi-profile -i TSGA061.bam -c TSGA061anvi.db --num-threads 10 --output-dir TSGA061_AnviProf --min-contig-length 100 --sample-name TSGA061 --cluster-contigs

anvi-run-scg-taxonomy -c TSGA061anvi.db

anvi-interactive -p ~/MollicuteGenomes2021/JGIMeta/TSGA061_AnviProf/PROFILE.db -c TSGA061anvi.db

#TSGA061_Bins

#ReRun NCBI meso lactu strain with prokka and try again.

#Outside of anvio

prokka --prefix PROKKA --outdir PROKKA_SRR6082029_fixed --cpus 5 SRR6082029_fixed.fa --gcode 4 --kingdom Bacteria

python3 gff_parser.py PROKKA_SRR6082029_fixed/PROKKA.gff --gene-calls SRR6082029_calls.txt --annotation SRR6082029_annot.txt

#In anvio

anvi-gen-contigs-database -f SRR6082029_fixed.fa -o SRR6082029anvi.db --external-gene-calls SRR6082029_calls.txt --ignore-internal-stop-codons -n anvitrySRR6082029

anvi-import-functions -c SRR6082029anvi.db -i SRR6082029_annot.txt

anvi-run-hmms -c SRR6082029anvi.db

bwa index ~/MollicuteGenomes2021/NCBIGenomeTest/SRR6082029_fixed.fa

bwa mem ~/MollicuteGenomes2021/NCBIGenomeTest/SRR6082029_fixed.fa SRR6082029.1_1.fastq SRR6082029.1_2.fastq > SRR6082029Aligned.sam

samtools index SRR6082029Aligned.sam

samtools fixmate -O bam SRR6082029Aligned.sam SRR6082029Aligned_fixmate.bam

samtools sort -o SRR6082029Aligned_sorted.bam -O bam -T temp SRR6082029Aligned_fixmate.bam

samtools index SRR6082029Aligned_sorted.bam

anvi-init-bam SRR6082029Aligned_sorted.bam -o SRR6082029.bam

anvi-profile -i SRR6082029.bam -c SRR6082029anvi.db --num-threads 10 --output-dir SRR6082029_AnviProf --min-contig-length 100 --sample-name SRR6082029 --cluster-contigs

anvi-run-scg-taxonomy -c SRR6082029anvi.db

anvi-interactive -p ~/MollicuteGenomes2021/NCBIGenomeTest/SRR6082029_AnviProf/PROFILE.db -c SRR6082029anvi.db

#SRR6082029_Bins

#Download fasta files from NCBI for reference genomes

#Anvio fix contig names

anvi-script-reformat-fasta Mesochaulicola.fasta -o Mesochaulicola-fixed.fa -l 0 --simplify-names

anvi-script-reformat-fasta Mesocorruscae.fasta -o Mesocorruscae-fixed.fa -l 0 --simplify-names

anvi-script-reformat-fasta Mesoentomophilum.fasta -o Mesoentomophilum-fixed.fa -l 0 --simplify-names

anvi-script-reformat-fasta Mesoflorum.fasta -o Mesoflorum-fixed.fa -l 0 --simplify-names

anvi-script-reformat-fasta Mesophoturis.fasta -o Mesophoturis-fixed.fa -l 0 --simplify-names

anvi-script-reformat-fasta Mesosyrphidae.fasta -o Mesosyrphidae-fixed.fa -l 0 --simplify-names

#Outside of anvio

prokka --prefix PROKKA --outdir PROKKA_Mesochaulicola --cpus 5 Mesochaulicola-fixed.fa --gcode 4 --kingdom Bacteria

python3 gff_parser.py PROKKA_Mesochaulicola/PROKKA.gff --gene-calls Mesochaulicola_calls.txt --annotation Mesochaulicola_annot.txt

prokka --prefix PROKKA --outdir PROKKA_Mesocorruscae --cpus 5 Mesocorruscae-fixed.fa --gcode 4 --kingdom Bacteria

python3 gff_parser.py PROKKA_Mesocorruscae/PROKKA.gff --gene-calls Mesocorruscae_calls.txt --annotation Mesocorruscae_annot.txt

prokka --prefix PROKKA --outdir PROKKA_Mesoentomophilum --cpus 5 Mesoentomophilum-fixed.fa --gcode 4 --kingdom Bacteria

python3 gff_parser.py PROKKA_Mesoentomophilum/PROKKA.gff --gene-calls Mesoentomophilum_calls.txt --annotation Mesoentomophilum_annot.txt

prokka --prefix PROKKA --outdir PROKKA_Mesoflorum --cpus 5 Mesoflorum-fixed.fa --gcode 4 --kingdom Bacteria

python3 gff_parser.py PROKKA_Mesoflorum/PROKKA.gff --gene-calls Mesoflorum_calls.txt --annotation Mesoflorum_annot.txt

prokka --prefix PROKKA --outdir PROKKA_Mesophoturis --cpus 5 Mesophoturis-fixed.fa --gcode 4 --kingdom Bacteria

python3 gff_parser.py PROKKA_Mesophoturis/PROKKA.gff --gene-calls Mesophoturis_calls.txt --annotation Mesophoturis_annot.txt

prokka --prefix PROKKA --outdir PROKKA_Mesosyrphidae --cpus 5 Mesosyrphidae-fixed.fa --gcode 4 --kingdom Bacteria

python3 gff_parser.py PROKKA_Mesosyrphidae/PROKKA.gff --gene-calls Mesosyrphidae_calls.txt --annotation Mesosyrphidae_annot.txt

#Back in anvio add in calls and make contigs database

anvi-gen-contigs-database -f Mesochaulicola-fixed.fa -o Mesochaulicolaanvi.db --external-gene-calls Mesochaulicola_calls.txt --ignore-internal-stop-codons -n anvitry_Mesochaulicola

anvi-import-functions -c Mesochaulicolaanvi.db -i Mesochaulicola_annot.txt

anvi-run-hmms -c Mesochaulicolaanvi.db --num-threads 10

anvi-gen-contigs-database -f Mesocorruscae-fixed.fa -o Mesocorruscaeanvi.db --external-gene-calls Mesocorruscae_calls.txt --ignore-internal-stop-codons -n anvitry_Mesocorruscae

anvi-import-functions -c Mesocorruscaeanvi.db -i Mesocorruscae_annot.txt

anvi-run-hmms -c Mesocorruscaeanvi.db --num-threads 10

anvi-gen-contigs-database -f Mesoentomophilum-fixed.fa -o Mesoentomophilumanvi.db --external-gene-calls Mesoentomophilum_calls.txt --ignore-internal-stop-codons -n anvitry_Mesoentomophilum

anvi-import-functions -c Mesoentomophilumanvi.db -i Mesoentomophilum_annot.txt

anvi-run-hmms -c Mesoentomophilumanvi.db --num-threads 10

anvi-gen-contigs-database -f Mesoflorum-fixed.fa -o Mesoflorumanvi.db --external-gene-calls Mesoflorum_calls.txt --ignore-internal-stop-codons -n anvitry_Mesoflorum

anvi-import-functions -c Mesoflorumanvi.db -i Mesoflorum_annot.txt

anvi-run-hmms -c Mesoflorumanvi.db --num-threads 10

anvi-gen-contigs-database -f Mesophoturis-fixed.fa -o Mesophoturisanvi.db --external-gene-calls Mesophoturis_calls.txt --ignore-internal-stop-codons -n anvitry_Mesophoturis

anvi-import-functions -c Mesophoturisanvi.db -i Mesophoturis_annot.txt

anvi-run-hmms -c Mesophoturisanvi.db --num-threads 10

anvi-gen-contigs-database -f Mesosyrphidae-fixed.fa -o Mesosyrphidaeanvi.db --external-gene-calls Mesosyrphidae_calls.txt --ignore-internal-stop-codons -n anvitry_Mesosyrphidae

anvi-import-functions -c Mesosyrphidaeanvi.db -i Mesosyrphidae_annot.txt

anvi-run-hmms -c Mesosyrphidaeanvi.db --num-threads 10

#Made a .txt file named ExternalGenomesNCBIandFiltEntAcro1.txt with the entacro file and the mesoplasmas from NCBI

#Found the file that spaountsiz used with the exact contigs needed. I have to redo everything with the new EntAcro1

anvi-script-reformat-fasta EntAcro1F.fasta -o EntAcro1F-fixed.fa -l 0 --simplify-names

prokka --prefix PROKKA --outdir PROKKA_EntAcro1F --cpus 5 EntAcro1F-fixed.fa --gcode 4 --kingdom Bacteria

python3 gff_parser.py PROKKA_EntAcro1F/PROKKA.gff --gene-calls EntAcro1F_calls.txt --annotation EntAcro1F_annot.txt

#Back in anvio add in calls and make contigs database

anvi-gen-contigs-database -f EntAcro1F-fixed.fa -o EntAcro1Fanvi.db --external-gene-calls EntAcro1F_calls.txt --ignore-internal-stop-codons -n anvitry_EntAcro1F

anvi-import-functions -c EntAcro1Fanvi.db -i EntAcro1F_annot.txt

anvi-run-hmms -c EntAcro1Fanvi.db --num-threads 10

#Make a pangenome

anvi-gen-genomes-storage -i InternalGenomeAllMAGsandNCBiMesoLact.txt -e ExternalGenomesNCBIandEntAcro1F.txt -o EntireMeso-GENOMES.db --gene-caller 'Prodigal'

#Leaving mcl-inflation at 7 because changing it to less strict didn't change anything from above

anvi-pan-genome -g EntireMeso-GENOMES.db -n EntireMeso_anvipan --output-dir EntireMeso_AnviPan --num-threads 10 --minbit 0.5 --mcl-inflation 7

anvi-display-pan -p EntireMeso_AnviPan/EntireMeso_anvipan-PAN.db -g EntireMeso-GENOMES.db

#EntireMeso-Bins

#Single_3

#EntireMeso_State

#Other_2

#Core_1

anvi-split -p EntireMeso_AnviPan/EntireMeso_anvipan-PAN.db -g EntireMeso-GENOMES.db -C EntireMeso_Bins -o Split_EntireMeso_Pans

anvi-get-sequences-for-gene-clusters -p Split_EntireMeso_Pans/Single_3/PAN.db -g EntireMeso-GENOMES.db --concatenate-gene-clusters -o EntireMeso_Single_3_SeqsPhylog.fa --max-num-genes-from-each-genome 1

FastTree -wag EntireMeso_Single_3_SeqsPhylog.fa > EntireMeso_Single_3_SeqsPhylog.tree

#Put .tree file into ITol.

#RUN WITH NUCLEOTIDES

anvi-get-sequences-for-gene-clusters -p Split_EntireMeso_Pans/Single_3/PAN.db -g EntireMeso-GENOMES.db --concatenate-gene-clusters -o EntireMeso_Single_3NuclPhylog.fa --report-DNA-sequences --max-num-genes-from-each-genome 1 --just-do-it

FastTree -nt EntireMeso_Single_3NuclPhylog.fa > EntireMeso_Single_3_NuclPhylog.tree

#To get txt files of all the gene info...

anvi-display-pan -p Split_EntireMeso_Pans/Single_3/PAN.db -g EntireMeso-GENOMES.db

#Bin_1 under Default

anvi-summarize -g EntireMeso-GENOMES.db -p Split_EntireMeso_Pans/Single_3/PAN.db -C default

anvi-display-pan -p Split_EntireMeso_Pans/Core_1/PAN.db -g EntireMeso-GENOMES.db

#Bin_1 under Default

anvi-summarize -g EntireMeso-GENOMES.db -p Split_EntireMeso_Pans/Core_1/PAN.db -C default

anvi-display-pan -p Split_EntireMeso_Pans/Other_2/PAN.db -g EntireMeso-GENOMES.db

#Bin_1 under Default

anvi-summarize -g EntireMeso-GENOMES.db -p Split_EntireMeso_Pans/Other_2/PAN.db -C default

#Adding average nucleotide identity: https://merenlab.org/tutorials/infant-gut/#adding-average-nucleotide-identity

anvi-compute-genome-similarity -i InternalGenomeAllMAGsandNCBiMesoLact.txt -e ExternalGenomesNCBIandEntAcro1F.txt -o EntireMeso-GENOMES.db --program pyANI -o EntireMeso_ANI -T 6 --pan-db EntireMeso_AnviPan/EntireMeso_anvipan-PAN.db

anvi-display-pan -p EntireMeso_AnviPan/EntireMeso_anvipan-PAN.db -g EntireMeso-GENOMES.db

#EntireMeso_ANI_State

#Just my genomes, MAGs, EntAcro1F and Lactucae

#Make a pangenome

anvi-gen-genomes-storage -i InternalGenomeAllMAGsandNCBiMesoLact.txt -e ExternalEntAcro1F.txt -o MyMeso-GENOMES.db --gene-caller 'Prodigal'

anvi-pan-genome -g MyMeso-GENOMES.db -n MyMeso_anvipan --output-dir MyMeso_AnviPan --num-threads 10 --minbit 0.5 --mcl-inflation 7

anvi-display-pan -p MyMeso_AnviPan/MyMeso_anvipan-PAN.db -g MyMeso-GENOMES.db

#MyMeso-Bins

#Single_3

#MyMeso_State

#Other_2

#Core_1

anvi-split -p MyMeso_AnviPan/EntireMeso_anvipan-PAN.db -g MyMeso-GENOMES.db -C MyMeso_Bins -o Split_MyMeso_Pans

anvi-get-sequences-for-gene-clusters -p Split_MyMeso_Pans/Single_3/PAN.db -g MyMeso-GENOMES.db --concatenate-gene-clusters -o MyMeso_Single_3_SeqsPhylog.fa --max-num-genes-from-each-genome 1

FastTree -wag MyMeso_Single_3_SeqsPhylog.fa > MyMeso_Single_3_SeqsPhylog.tree

#Put .tree file into ITol.

#RUN WITH NUCLEOTIDES

anvi-get-sequences-for-gene-clusters -p Split_MyMeso_Pans/Single_3/PAN.db -g MyMeso-GENOMES.db --concatenate-gene-clusters -o MyMeso_Single_3NuclPhylog.fa --report-DNA-sequences --max-num-genes-from-each-genome 1 --just-do-it

FastTree -nt MyMeso_Single_3NuclPhylog.fa > MyMeso_Single_3_NuclPhylog.tree

#To get txt files of all the gene info...

anvi-display-pan -p Split_MyMeso_Pans/Single_3/PAN.db -g MyMeso-GENOMES.db

#Bin_1 under Default

anvi-summarize -g MyMeso-GENOMES.db -p Split_MyMeso_Pans/Single_3/PAN.db -C default

anvi-display-pan -p Split_MyMeso_Pans/Core_1/PAN.db -g MyMeso-GENOMES.db

#Bin_1 under Default

anvi-summarize -g MyMeso-GENOMES.db -p Split_MyMeso_Pans/Core_1/PAN.db -C default

anvi-display-pan -p Split_MyMeso_Pans/Other_2/PAN.db -g MyMeso-GENOMES.db

#Bin_1 under Default

anvi-summarize -g MyMeso-GENOMES.db -p Split_MyMeso_Pans/Other_2/PAN.db -C default

#Try to use a lower mcl inflation number to confirm...

#Using the same files...just name the -GENOMES.db differently

anvi-gen-genomes-storage -i InternalGenomeandTopJGIMAGsandNCBiMesoLact.txt -e ExternalFilt3EntAcro1Genome.txt -o MesoandMAG3-GENOMES.db --gene-caller 'Prodigal'

#Changed mcl-inflation value

anvi-pan-genome -g MesoandMAG3-GENOMES.db -n MesoandMAG3_anvipan --output-dir MesoandMAG3_AnviPan --num-threads 10 --minbit 0.5 --mcl-inflation 3

anvi-display-pan -p MesoandMAG3_AnviPan/MesoandMAG3_anvipan-PAN.db -g MesoandMAG3-GENOMES.db

#MesoandMAG3_Bins

#Core_1 & Other_2 & Single_3

#MesoandMAG3_State

#SPIROPLASMA GENOMES

mkdir FastQC

fastqc ~/MollicuteGenomes2021/SpiroplasmaGenomes/*.fastq -o ~/MollicuteGenomes2021/SpiroplasmaGenomes/FastQC/

#Need to remove adapters, use trimmomatic

#MUST MOVE ADAPTER FILE TO DIRECTORY YOU WORK IN

cp /usr/local/bioinf_tools/Trimmomatic-0.39/adapters/TruSeq2-PE.fa ~/MollicuteGenomes2021/SpiroplasmaGenomes/

java -jar /usr/local/bioinf_tools/Trimmomatic-0.39/trimmomatic-0.39.jar PE -threads 8 -phred33 210727JKS002669_S106_L001_R1_001.fastq 210727JKS002669_S106_L001_R2_001.fastq JKS002669_R1_paired.fastq JKS002669_R1_unpaired.fastq JKS002669_R2_paired.fastq JKS002669_R2_unpaired.fastq ILLUMINACLIP:TruSeq2-PE.fa:2:30:10 LEADING:3 TRAILING:3 SLIDINGWINDOW:4:15 MINLEN:36

java -jar /usr/local/bioinf_tools/Trimmomatic-0.39/trimmomatic-0.39.jar PE -threads 8 -phred33 210727JKS002670_S107_L001_R1_001.fastq 210727JKS002670_S107_L001_R2_001.fastq JKS002670_R1_paired.fastq JKS002670_R1_unpaired.fastq JKS002670_R2_paired.fastq JKS002670_R2_unpaired.fastq ILLUMINACLIP:TruSeq2-PE.fa:2:30:10 LEADING:3 TRAILING:3 SLIDINGWINDOW:4:15 MINLEN:36

java -jar /usr/local/bioinf_tools/Trimmomatic-0.39/trimmomatic-0.39.jar PE -threads 8 -phred33 210727JKS002670x2_S108_L001_R1_001.fastq 210727JKS002670x2_S108_L001_R2_001.fastq JKS002670x2_R1_paired.fastq JKS002670x2_R1_unpaired.fastq JKS002670x2_R2_paired.fastq JKS002670x2_R2_unpaired.fastq ILLUMINACLIP:TruSeq2-PE.fa:2:30:10 LEADING:3 TRAILING:3 SLIDINGWINDOW:4:15 MINLEN:36

java -jar /usr/local/bioinf_tools/Trimmomatic-0.39/trimmomatic-0.39.jar PE -threads 8 -phred33 210727JKS002671_S109_L001_R1_001.fastq 210727JKS002671_S109_L001_R2_001.fastq JKS002671_R1_paired.fastq JKS002671_R1_unpaired.fastq JKS002671_R2_paired.fastq JKS002671_R2_unpaired.fastq ILLUMINACLIP:TruSeq2-PE.fa:2:30:10 LEADING:3 TRAILING:3 SLIDINGWINDOW:4:15 MINLEN:36

java -jar /usr/local/bioinf_tools/Trimmomatic-0.39/trimmomatic-0.39.jar PE -threads 8 -phred33 210727JKS002671x2_S110_L001_R1_001.fastq 210727JKS002671x2_S110_L001_R2_001.fastq JKS002671x2_R1_paired.fastq JKS002671x2_R1_unpaired.fastq JKS002671x2_R2_paired.fastq JKS002671x2_R2_unpaired.fastq ILLUMINACLIP:TruSeq2-PE.fa:2:30:10 LEADING:3 TRAILING:3 SLIDINGWINDOW:4:15 MINLEN:36

#Run Fastqc

fastqc *_paired.fastq -o FastQC/

#unicycler

unicycler -1 JKS002669_R1_paired.fastq -2 JKS002669_R2_paired.fastq -o JKS002669_unicycler -t 20

unicycler -1 JKS002670x2_R1_paired.fastq -2 JKS002670x2_R2_paired.fastq -o JKS002670x2_unicycler -t 20

unicycler -1 JKS002671x2_R1_paired.fastq -2 JKS002671x2_R2_paired.fastq -o JKS002671x2_unicycler -t 20

mkdir QuastOutput

quast.py ~/MollicuteGenomes2021/SpiroplasmaGenomes/JKS002669_unicycler/JKS002669_assembly.fasta -o ~/MollicuteGenomes2021/SpiroplasmaGenomes/QuastOutput/JKS002669_unicycler

quast.py ~/MollicuteGenomes2021/SpiroplasmaGenomes/JKS002670_unicycler/JKS002670_assembly.fasta -o ~/MollicuteGenomes2021/SpiroplasmaGenomes/QuastOutput/JKS002670_unicycler

quast.py ~/MollicuteGenomes2021/SpiroplasmaGenomes/JKS002670x2_unicycler/JKS002670x2_assembly.fasta -o ~/MollicuteGenomes2021/SpiroplasmaGenomes/QuastOutput/JKS002670x2_unicycler

quast.py ~/MollicuteGenomes2021/SpiroplasmaGenomes/JKS002671_unicycler/JKS002671_assembly.fasta -o ~/MollicuteGenomes2021/SpiroplasmaGenomes/QuastOutput/JKS002671_unicycler

quast.py ~/MollicuteGenomes2021/SpiroplasmaGenomes/JKS002671x2_unicycler/JKS002671x2_assembly.fasta -o ~/MollicuteGenomes2021/SpiroplasmaGenomes/QuastOutput/JKS002671x2_unicycler

#Blasted all the contigs and removed contamination. Now running quast on "filtered" genomes to see what they look like.

quast.py ~/MollicuteGenomes2021/SpiroplasmaGenomes/FiltJKS002669.fasta -o ~/MollicuteGenomes2021/SpiroplasmaGenomes/QuastOutput/FiltJKS002669

quast.py ~/MollicuteGenomes2021/SpiroplasmaGenomes/FiltJKS002670x2.fasta -o ~/MollicuteGenomes2021/SpiroplasmaGenomes/QuastOutput/FiltJKS002670x2

quast.py ~/MollicuteGenomes2021/SpiroplasmaGenomes/FiltJKS002671x2.fasta -o ~/MollicuteGenomes2021/SpiroplasmaGenomes/QuastOutput/FiltJKS002671x2

#Run busco on xanadu

busco -m genome -i FiltJKS002669.fasta -o FiltJKS002669_busco -l /isg/shared/databases/BUSCO/odb10/lineages/tenericutes_odb10/

busco -m genome -i FiltJKS002670.fasta -o FiltJKS002670_busco -l /isg/shared/databases/BUSCO/odb10/lineages/tenericutes_odb10/

busco -m genome -i FiltJKS002670x2.fasta -o FiltJKS002670x2_busco -l /isg/shared/databases/BUSCO/odb10/lineages/tenericutes_odb10/

busco -m genome -i FiltJKS002671.fasta -o FiltJKS002671_busco -l /isg/shared/databases/BUSCO/odb10/lineages/tenericutes_odb10/

busco -m genome -i FiltJKS002671x2.fasta -o FiltJKS002671x2_busco -l /isg/shared/databases/BUSCO/odb10/lineages/tenericutes_odb10/

busco -m genome -i EntAcro10F.fasta -o EntAcro10F_busco -l /isg/shared/databases/BUSCO/odb10/lineages/tenericutes_odb10/

#I will need to include S.platyhelix in this study.

#Spiro genomes and platyhelix only

#completely restart with my genomes.

#Load anvio

anvi-script-reformat-fasta /home/egreen/MollicuteGenomes2021/SpiroplasmaGenomes/SpiroAnvio/FiltJKS002669.fasta -o FiltJKS002669-fixed.fa -l 0 --simplify-names -r Keep2669tabs

anvi-script-reformat-fasta /home/egreen/MollicuteGenomes2021/SpiroplasmaGenomes/SpiroAnvio/FiltJKS002670x2.fasta -o FiltJKS002670x2-fixed.fa -l 0 --simplify-names -r Keep2670x2tabs

anvi-script-reformat-fasta /home/egreen/MollicuteGenomes2021/SpiroplasmaGenomes/SpiroAnvio/FiltJKS002671x2.fasta -o FiltJKS002671x2-fixed.fa -l 0 --simplify-names -r Keep2671x2tabs

anvi-script-reformat-fasta /home/egreen/MollicuteGenomes2021/SpiroplasmaGenomes/SpiroAnvio/Splatyhelix.fasta -o Splatyhelix-fixed.fa -l 0 --simplify-names -r KeepSplatyhelixtabs

#Outside of anvio

prokka --prefix PROKKA --outdir PROKKA_JKS002669-fixed --cpus 5 FiltJKS002669-fixed.fa --gcode 4 --kingdom Bacteria

python3 gff_parser.py PROKKA_JKS002669-fixed/PROKKA.gff --gene-calls FiltJKS002669gene_calls.txt --annotation FiltJKS002669gene_annot.txt

prokka --prefix PROKKA --outdir PROKKA_JKS002670x2-fixed --cpus 5 FiltJKS002670x2-fixed.fa --gcode 4 --kingdom Bacteria

python3 gff_parser.py PROKKA_JKS002670x2-fixed/PROKKA.gff --gene-calls FiltJKS002670x2gene_calls.txt --annotation FiltJKS002670x2gene_annot.txt

prokka --prefix PROKKA --outdir PROKKA_JKS002671x2-fixed --cpus 5 FiltJKS002671x2-fixed.fa --gcode 4 --kingdom Bacteria

python3 gff_parser.py PROKKA_JKS002671x2-fixed/PROKKA.gff --gene-calls FiltJKS002671x2gene_calls.txt --annotation FiltJKS002671x2gene_annot.txt

prokka --prefix PROKKA --outdir PROKKA_Splatyhelix-fixed --cpus 5 Splatyhelix-fixed.fa --gcode 4 --kingdom Bacteria

python3 gff_parser.py PROKKA_Splatyhelix-fixed/PROKKA.gff --gene-calls Splatyhelixgene_calls.txt --annotation Splatyhelixgene_annot.txt

#Load anvio again

anvi-gen-contigs-database -f FiltJKS002669-fixed.fa -o FiltJKS002669anvi.db --external-gene-calls FiltJKS002669gene_calls.txt --ignore-internal-stop-codons -n anvitry2669

anvi-import-functions -c FiltJKS002669anvi.db -i FiltJKS002669gene_annot.txt

anvi-run-hmms -c FiltJKS002669anvi.db

bwa index ~/MollicuteGenomes2021/SpiroplasmaGenomes/SpiroAnvio/FiltJKS002669-fixed.fa

bwa mem ~/MollicuteGenomes2021/SpiroplasmaGenomes/SpiroAnvio/FiltJKS002669-fixed.fa ~/MollicuteGenomes2021/SpiroplasmaGenomes/JKS002669_R1_paired.fastq ~/MollicuteGenomes2021/SpiroplasmaGenomes/JKS002669_R2_paired.fastq > FiltJKS002669Aligned.sam -t 10

samtools index FiltJKS002669Aligned.sam

samtools fixmate -O bam FiltJKS002669Aligned.sam FiltJKS002669Aligned_fixmate.bam

samtools sort -o FiltJKS002669Aligned_sorted.bam -O bam -T temp FiltJKS002669Aligned_fixmate.bam

samtools index FiltJKS002669Aligned_sorted.bam

anvi-init-bam FiltJKS002669Aligned_sorted.bam -o FiltJKS002669.bam

anvi-run-scg-taxonomy -c FiltJKS002669anvi.db

anvi-profile -i FiltJKS002669.bam -c FiltJKS002669anvi.db --num-threads 10 --output-dir FiltJKS002669_AnviProf --min-contig-length 100 --sample-name FiltJKS002669 --cluster-contigs

anvi-interactive -p ~/MollicuteGenomes2021/SpiroplasmaGenomes/SpiroAnvio/FiltJKS002669_AnviProf/PROFILE.db -c FiltJKS002669anvi.db

#FiltJKS002669_Bins, Bin_1

anvi-gen-contigs-database -f FiltJKS002670x2-fixed.fa -o FiltJKS002670x2anvi.db --external-gene-calls FiltJKS002670x2gene_calls.txt --ignore-internal-stop-codons -n anvitry2670x2

anvi-import-functions -c FiltJKS002670x2anvi.db -i FiltJKS002670x2gene_annot.txt

anvi-run-hmms -c FiltJKS002670x2anvi.db

bwa index ~/MollicuteGenomes2021/SpiroplasmaGenomes/SpiroAnvio/FiltJKS002670x2-fixed.fa

bwa mem ~/MollicuteGenomes2021/SpiroplasmaGenomes/SpiroAnvio/FiltJKS002670x2-fixed.fa ~/MollicuteGenomes2021/SpiroplasmaGenomes/JKS002670x2_R1_paired.fastq ~/MollicuteGenomes2021/SpiroplasmaGenomes/JKS002670x2_R2_paired.fastq > FiltJKS002670x2Aligned.sam -t 10

samtools index FiltJKS002670x2Aligned.sam

samtools fixmate -O bam FiltJKS002670x2Aligned.sam FiltJKS002670x2Aligned_fixmate.bam

samtools sort -o FiltJKS002670x2Aligned_sorted.bam -O bam -T temp FiltJKS002670x2Aligned_fixmate.bam

samtools index FiltJKS002670x2Aligned_sorted.bam

anvi-init-bam FiltJKS002670x2Aligned_sorted.bam -o FiltJKS002670x2.bam

anvi-run-scg-taxonomy -c FiltJKS002670x2anvi.db

anvi-profile -i FiltJKS002670x2.bam -c FiltJKS002670x2anvi.db --num-threads 10 --output-dir FiltJKS002670x2_AnviProf --min-contig-length 100 --sample-name FiltJKS002670x2 --cluster-contigs

anvi-interactive -p ~/MollicuteGenomes2021/SpiroplasmaGenomes/SpiroAnvio/FiltJKS002670x2_AnviProf/PROFILE.db -c FiltJKS002670x2anvi.db

#FiltJKS002670x2_Bins, Bin_1

anvi-gen-contigs-database -f FiltJKS002671x2-fixed.fa -o FiltJKS002671x2anvi.db --external-gene-calls FiltJKS002671x2gene_calls.txt --ignore-internal-stop-codons -n anvitry2671x2

anvi-import-functions -c FiltJKS002671x2anvi.db -i FiltJKS002671x2gene_annot.txt

anvi-run-hmms -c FiltJKS002671x2anvi.db

bwa index ~/MollicuteGenomes2021/SpiroplasmaGenomes/SpiroAnvio/FiltJKS002671x2-fixed.fa

bwa mem ~/MollicuteGenomes2021/SpiroplasmaGenomes/SpiroAnvio/FiltJKS002671x2-fixed.fa ~/MollicuteGenomes2021/SpiroplasmaGenomes/JKS002671x2_R1_paired.fastq ~/MollicuteGenomes2021/SpiroplasmaGenomes/JKS002671x2_R2_paired.fastq > FiltJKS002671x2Aligned.sam -t 10

samtools index FiltJKS002671x2Aligned.sam

samtools fixmate -O bam FiltJKS002671x2Aligned.sam FiltJKS002671x2Aligned_fixmate.bam

samtools sort -o FiltJKS002671x2Aligned_sorted.bam -O bam -T temp FiltJKS002671x2Aligned_fixmate.bam

samtools index FiltJKS002671x2Aligned_sorted.bam

anvi-init-bam FiltJKS002671x2Aligned_sorted.bam -o FiltJKS002671x2.bam

anvi-run-scg-taxonomy -c FiltJKS002671x2anvi.db

anvi-profile -i FiltJKS002671x2.bam -c FiltJKS002671x2anvi.db --num-threads 10 --output-dir FiltJKS002671x2_AnviProf --min-contig-length 100 --sample-name FiltJKS002671x2 --cluster-contigs

anvi-interactive -p ~/MollicuteGenomes2021/SpiroplasmaGenomes/SpiroAnvio/FiltJKS002671x2_AnviProf/PROFILE.db -c FiltJKS002671x2anvi.db

#Contig 3 and 4 have high GC, 48%

#FiltJKS002671x2_Bins, Bin_1

#"External Genomes"

anvi-gen-contigs-database -f Splatyhelix-fixed.fa -o Splatyhelixanvi.db --external-gene-calls Splatyhelixgene_calls.txt --ignore-internal-stop-codons -n anvitrySplatyhelix

anvi-import-functions -c Splatyhelixanvi.db -i Splatyhelixgene_annot.txt

anvi-run-hmms -c Splatyhelixanvi.db

#Found the file that spaountsiz used with the exact contigs needed. I have to redo everything with the new EntAcro10

anvi-script-reformat-fasta EntAcro10F.fasta -o EntAcro10F-fixed.fa -l 0 --simplify-names

prokka --prefix PROKKA --outdir PROKKA_EntAcro10F --cpus 5 EntAcro10F-fixed.fa --gcode 4 --kingdom Bacteria

python3 gff_parser.py PROKKA_EntAcro10F/PROKKA.gff --gene-calls EntAcro10F_calls.txt --annotation EntAcro10F_annot.txt

#Back in anvio add in calls and make contigs database

anvi-gen-contigs-database -f EntAcro10F-fixed.fa -o EntAcro10Fanvi.db --external-gene-calls EntAcro10F_calls.txt --ignore-internal-stop-codons -n anvitry_EntAcro10F

anvi-import-functions -c EntAcro10Fanvi.db -i EntAcro10F_annot.txt

anvi-run-hmms -c EntAcro10Fanvi.db --num-threads 10

anvi-gen-genomes-storage -i InternalSpiro.txt -e ExternalGenomesPlatyhelixEntAcro10.txt -o Spiro-GENOMES.db --gene-caller 'Prodigal'

anvi-pan-genome -g Spiro-GENOMES.db -n Spiro_anvipan --output-dir Spiro_AnviPan --num-threads 10 --minbit 0.5 --mcl-inflation 7

anvi-display-pan -p Spiro_AnviPan/Spiro_anvipan-PAN.db -g Spiro-GENOMES.db

#Spiro_Bins

#Core_1

#Other_2

# Single_3

#472 total SCG,

#RandomSingle_4 includes 100 random gene clusters I selected from the single copy gene cluster

#Spiro_state

anvi-split -p Spiro_AnviPan/Spiro_anvipan-PAN.db -g Spiro-GENOMES.db -C Spiro_Bins -o Split_Spiro_Pans

anvi-display-pan -p Split_Spiro_Pans/RandomSingle_4/PAN.db -g Spiro-GENOMES.db

#Bin_1 under Default

anvi-summarize -g Spiro-GENOMES.db -p Split_Spiro_Pans/RandomSingle_4/PAN.db -C default

anvi-display-pan -p Split_Spiro_Pans/Single_3/PAN.db -g Spiro-GENOMES.db

#Bin_1 under Default

anvi-summarize -g Spiro-GENOMES.db -p Split_Spiro_Pans/Single_3/PAN.db -C default

anvi-display-pan -p Split_Spiro_Pans/Core_1/PAN.db -g Spiro-GENOMES.db

#Bin_1 and Bin_2 under Default, split them by the dendrogram at the top, some are just ant genes, Bin_2 and other are all the seqs Bin_1

anvi-summarize -g Spiro-GENOMES.db -p Split_Spiro_Pans/Core_1/PAN.db -C default

anvi-display-pan -p Split_Spiro_Pans/Other_2/PAN.db -g Spiro-GENOMES.db

#Bin_1 and Bin_2 under Default split them based on what was only in single genomes Bin_1 and then overlapping between them Bin_2

anvi-summarize -g Spiro-GENOMES.db -p Split_Spiro_Pans/Other_2/PAN.db -C default

anvi-get-sequences-for-gene-clusters -p Split_Spiro_Pans/RandomSingle_4/PAN.db -g Spiro-GENOMES.db --concatenate-gene-clusters -o Spiro_RandomSingle_4_SeqsPhylog.fa --max-num-genes-from-each-genome 1

FastTree -wag Spiro_RandomSingle_4_SeqsPhylog.fa > Spiro_RandomSingle_4_SeqsPhylog.tree

#RUN WITH NUCLEOTIDES

anvi-get-sequences-for-gene-clusters -p Split_Spiro_Pans/RandomSingle_4/PAN.db -g Spiro-GENOMES.db --concatenate-gene-clusters -o Spiro_RandomSingle_4NuclPhylog.fa --report-DNA-sequences --max-num-genes-from-each-genome 1 --just-do-it

FastTree -nt Spiro_RandomSingle_4NuclPhylog.fa > Spiro_RandomSingle_4NuclPhylog.tree

#Adding in Spiroplasma genomes from NCBI

#in SpiroNCBI directory

#load anvio

anvi-script-reformat-fasta /home/egreen/MollicuteGenomes2021/SpiroplasmaGenomes/SpiroNCBI/Scitri.fasta -o Scitri-fixed.fa -l 0 --simplify-names -r KeepScitritabs

anvi-script-reformat-fasta /home/egreen/MollicuteGenomes2021/SpiroplasmaGenomes/SpiroNCBI/Seriocheiris.fasta -o Seriocheiris-fixed.fa -l 0 --simplify-names -r KeepSeriocheiris

anvi-script-reformat-fasta /home/egreen/MollicuteGenomes2021/SpiroplasmaGenomes/SpiroNCBI/Ssabaudiense.fasta -o Ssabaudiense-fixed.fa -l 0 --simplify-names -r KeepSsabaudiense

anvi-script-reformat-fasta /home/egreen/MollicuteGenomes2021/SpiroplasmaGenomes/SpiroNCBI/Spoulsonii.fasta -o Spoulsonii-fixed.fa -l 0 --simplify-names -r KeepSpoulsonii

anvi-script-reformat-fasta /home/egreen/MollicuteGenomes2021/SpiroplasmaGenomes/SpiroNCBI/Sapis.fasta -o Sapis-fixed.fa -l 0 --simplify-names -r KeepSapis

anvi-script-reformat-fasta /home/egreen/MollicuteGenomes2021/SpiroplasmaGenomes/SpiroNCBI/Sdiminutum.fasta -o Sdiminutum-fixed.fa -l 0 --simplify-names -r KeepSdiminutum

anvi-script-reformat-fasta /home/egreen/MollicuteGenomes2021/SpiroplasmaGenomes/SpiroNCBI/Shelicoides.fasta -o Shelicoides-fixed.fa -l 0 --simplify-names -r KeepShelicoides

prokka --prefix PROKKA --outdir PROKKA_Scitri-fixed --cpus 5 Scitri-fixed.fa --gcode 4 --kingdom Bacteria

python3 gff_parser.py PROKKA_Scitri-fixed/PROKKA.gff --gene-calls Scitrigene_calls.txt --annotation Scitrigene_annot.txt

prokka --prefix PROKKA --outdir PROKKA_Seriocheiris-fixed --cpus 5 Seriocheiris-fixed.fa --gcode 4 --kingdom Bacteria

python3 gff_parser.py PROKKA_Seriocheiris-fixed/PROKKA.gff --gene-calls Seriocheirisgene_calls.txt --annotation Seriocheirisgene_annot.txt

prokka --prefix PROKKA --outdir PROKKA_Ssabaudiense-fixed --cpus 5 Ssabaudiense-fixed.fa --gcode 4 --kingdom Bacteria

python3 gff_parser.py PROKKA_Ssabaudiense-fixed/PROKKA.gff --gene-calls Ssabaudiensegene_calls.txt --annotation Ssabaudiensegene_annot.txt

prokka --prefix PROKKA --outdir PROKKA_Spoulsonii-fixed --cpus 5 Spoulsonii-fixed.fa --gcode 4 --kingdom Bacteria

python3 gff_parser.py PROKKA_Spoulsonii-fixed/PROKKA.gff --gene-calls Spoulsoniigene_calls.txt --annotation Spoulsoniigene_annot.txt

prokka --prefix PROKKA --outdir PROKKA_Sapis-fixed --cpus 5 Sapis-fixed.fa --gcode 4 --kingdom Bacteria

python3 gff_parser.py PROKKA_Sapis-fixed/PROKKA.gff --gene-calls Sapisgene_calls.txt --annotation Sapisgene_annot.txt

prokka --prefix PROKKA --outdir PROKKA_Sdiminutum-fixed --cpus 5 Sdiminutum-fixed.fa --gcode 4 --kingdom Bacteria

python3 gff_parser.py PROKKA_Sdiminutum-fixed/PROKKA.gff --gene-calls Sdiminutumgene_calls.txt --annotation Sdiminutumgene_annot.txt

prokka --prefix PROKKA --outdir PROKKA_Shelicoides-fixed --cpus 5 Shelicoides-fixed.fa --gcode 4 --kingdom Bacteria

python3 gff_parser.py PROKKA_Shelicoides-fixed/PROKKA.gff --gene-calls Shelicoidesgene_calls.txt --annotation Shelicoidesgene_annot.txt

#In anvio

anvi-gen-contigs-database -f Scitri-fixed.fa -o Scitrianvi.db --external-gene-calls Scitrigene_calls.txt --ignore-internal-stop-codons -n anvitryScitri

anvi-import-functions -c Scitrianvi.db -i Scitrigene_annot.txt

anvi-run-hmms -c Scitrianvi.db

anvi-gen-contigs-database -f Seriocheiris-fixed.fa -o Seriocheirisanvi.db --external-gene-calls Seriocheirisgene_calls.txt --ignore-internal-stop-codons -n anvitrySeriocheiris

anvi-import-functions -c Seriocheirisanvi.db -i Seriocheirisgene_annot.txt

anvi-run-hmms -c Seriocheirisanvi.db

anvi-gen-contigs-database -f Ssabaudiense-fixed.fa -o Ssabaudienseanvi.db --external-gene-calls Ssabaudiensegene_calls.txt --ignore-internal-stop-codons -n anvitrySsabaudiense

anvi-import-functions -c Ssabaudienseanvi.db -i Ssabaudiensegene_annot.txt

anvi-run-hmms -c Ssabaudienseanvi.db

anvi-gen-contigs-database -f Spoulsonii-fixed.fa -o Spoulsoniianvi.db --external-gene-calls Spoulsoniigene_calls.txt --ignore-internal-stop-codons -n anvitrySpoulsonii

anvi-import-functions -c Spoulsoniianvi.db -i Spoulsoniigene_annot.txt

anvi-run-hmms -c Spoulsoniianvi.db

anvi-gen-contigs-database -f Sapis-fixed.fa -o Sapisanvi.db --external-gene-calls Sapisgene_calls.txt --ignore-internal-stop-codons -n anvitrySapis

anvi-import-functions -c Sapisanvi.db -i Sapisgene_annot.txt

anvi-run-hmms -c Sapisanvi.db

anvi-gen-contigs-database -f Sdiminutum-fixed.fa -o Sdiminutumanvi.db --external-gene-calls Sdiminutumgene_calls.txt --ignore-internal-stop-codons -n anvitrySdiminutum

anvi-import-functions -c Sdiminutumanvi.db -i Sdiminutumgene_annot.txt

anvi-run-hmms -c Sdiminutumanvi.db

anvi-gen-contigs-database -f Shelicoides-fixed.fa -o Shelicoidesanvi.db --external-gene-calls Shelicoidesgene_calls.txt --ignore-internal-stop-codons -n anvitryShelicoides

anvi-import-functions -c Shelicoidesanvi.db -i Shelicoidesgene_annot.txt

anvi-run-hmms -c Shelicoidesanvi.db

anvi-gen-genomes-storage -i InternalSpiro.txt -e ExternalGenomesNCBIPlatyhelixEntAcro10.txt -o SpiroNCBI-GENOMES.db --gene-caller 'Prodigal'

anvi-pan-genome -g SpiroNCBI-GENOMES.db -n SpiroNCBI_anvipan --output-dir SpiroNCBI_AnviPan --num-threads 10 --minbit 0.5 --mcl-inflation 7

anvi-display-pan -p SpiroNCBI_AnviPan/SpiroNCBI_anvipan-PAN.db -g SpiroNCBI-GENOMES.db

#SpiroNCBI_Bins

#Core_1

#Other_2

#Single_3- 80 gene clusters

#SpiroNCBI_State

anvi-split -p SpiroNCBI_AnviPan/SpiroNCBI_anvipan-PAN.db -g SpiroNCBI-GENOMES.db -C SpiroNCBI_Bins -o Split_SpiroNCBI_Pans

anvi-display-pan -p Split_SpiroNCBI_Pans/Single_3/PAN.db -g SpiroNCBI-GENOMES.db

#Bin_1 under Default

anvi-summarize -g SpiroNCBI-GENOMES.db -p Split_SpiroNCBI_Pans/Single_3/PAN.db -C default

#For Tree

anvi-get-sequences-for-gene-clusters -p Split_SpiroNCBI_Pans/Single_3/PAN.db -g SpiroNCBI-GENOMES.db --concatenate-gene-clusters -o SpiroNCBI_Single_3_SeqsPhylog.fa --max-num-genes-from-each-genome 1

FastTree -wag SpiroNCBI_Single_3_SeqsPhylog.fa > SpiroNCBI_Single_3_SeqsPhylog.tree

#Put .tree file into ITol.

#Nucleotides

anvi-get-sequences-for-gene-clusters -p Split_SpiroNCBI_Pans/Single_3/PAN.db -g SpiroNCBI-GENOMES.db --concatenate-gene-clusters -o SpiroNCBI_Single_3_NuclPhylog.fa --max-num-genes-from-each-genome 1 --report-DNA-sequences --just-do-it

FastTree -nt SpiroNCBI_Single_3_NuclPhylog.fa > SpiroNCBI_Single_3_NuclPhylog.tree

#Adding average nucleotide identity: https://merenlab.org/tutorials/infant-gut/#adding-average-nucleotide-identity

anvi-compute-genome-similarity -i InternalSpiro.txt -e ExternalGenomesNCBIPlatyhelixEntAcro10.txt -o SpiroNCBI-GENOMES.db --program pyANI -o SpiroNCBI_ANI -T 6 --pan-db SpiroNCBI_AnviPan/SpiroNCBI_anvipan-PAN.db

anvi-display-pan -p SpiroNCBI_AnviPan/SpiroNCBI_anvipan-PAN.db -g SpiroNCBI-GENOMES.db

SpiroNCBI_ANI_State

#Look at SpiroNCBI_ANI to get txt file of all the percentages.

2-8-2022

#Adding more "core" genes to the phylogeny.

#Making a pangenome and naming it "Feb8Test", it has all MAGS, EntAcro1, My meso genomes and M. lactucae (mine & NCBI)

#Make a pangenome

anvi-gen-genomes-storage -i InternalGenomeAllMAGsandNCBiMesoLact.txt -e ExternalEntAcro1F.txt -o Feb8Test-GENOMES.db --gene-caller 'Prodigal'

#Change mcl-inflation from 7 to 3. It is less strict, but I'm not sure if that matters

anvi-pan-genome -g Feb8Test-GENOMES.db -n Feb8Test_anvipan --output-dir Feb8Test_AnviPan --num-threads 10 --minbit 0.5 --mcl-inflation 3

anvi-display-pan -p Feb8Test_AnviPan/Feb8Test_anvipan-PAN.db -g Feb8Test-GENOMES.db

#BinCollection Named Core_Bin_1

#Bin also named Core_Bin_1

#Use "Search" tab, then "Seach gene clusters using filters"

#Min number of genomes gene cluster occurs= 18 & Max functional homogeneity index= .99 total of 550 gene clusters

anvi-split -p Feb8Test_AnviPan/Feb8Test_anvipan-PAN.db -g Feb8Test-GENOMES.db -C Core_Bin_1 -o Split_Feb8Test_Pans

anvi-get-sequences-for-gene-clusters -p Split_Feb8Test_Pans/Core_Bin_1/PAN.db -g Feb8Test-GENOMES.db --concatenate-gene-clusters -o Feb8Test_Core_Bin_1_SeqsPhylog.fa --max-num-genes-from-each-genome 1

FastTree -wag Feb8Test_Core_Bin_1_SeqsPhylog.fa > Feb8Test_Core_Bin_1_SeqsPhylog.tree

#Do not concatenate gene clusters. Try to get separate files for the gene clusters and orf core can do it for me

anvi-get-sequences-for-gene-clusters -p Split_Feb8Test_Pans/Core_Bin_1/PAN.db -g Feb8Test-GENOMES.db -o Feb8Test_Core_Bin_1_SeqsPhylog.fa --max-num-genes-from-each-genome 1

#530/550 gene clusters passed

#The fasta file had all the gene listed separately for each genome. this equals ~11k instances?

#How do i get each gene cluster into their own file?

#Now I can use orfcore

#Feb 22 2022

#Talked to JK, I need to do step 1, and then can skip to step 4.

#MAKE SURE TO CLOSE ANVIO

perl Convert_anvio_mult_faa_to_family_faas.pl -i Feb8Test_Core_Bin_1_SeqsPhylog.fa -o Feb8TestMesoFaasOutput

#1) Obtain a list of fasta files for the target protein(s) to be used to construct the HMMs with which the target genomes will be searched. Each target protein should have its own fasta file.Make a list of these files as input for step 2 (e.g., ls *.faa > fastalist).

#In ORFcore directory:

ls ~/MollicuteGenomes2021/Feb8TestMesoFaasOutput/*.faa > Feb8TestMesoFastaList

#4)Correct anomalous sequences, trimming ends if too long, adding X's if too short or replacing potential chimeras with X's. Produces a series of multiple alignments,one for each protein type. Note: stop codons are excluded for nucleotide data because they are included inconsistantly in gene files, i.e., the outputted corrected genes will lack these codons (which might not be conserved throughput the dataset anyway).

ORFcor.pl

DEPENDANCIES: BLAST+ suite, MUSCLE, Perl "Parallel::ForkManager" module, EMBOSS "transeq" if using nucleotides as input data

#made Feb8MesoORFcor directory to keep everything together

#Run in ~/MollicuteGenomes2021/Feb8MesoORFcor

perl ~/ORFcor/ORFcor.pl -i ~/ORFcor/Feb8TestMesoFastaList

#made the directory ORFcor_multiple_sequence_files

#5) Wrapper script to make multiple sequence alignments for each corrected sequence type using MUSCLE.

Mult_MUSCLE.pl

DEPENDANCIES: BLAST+ suite, MUSCLE, Perl \"Parallel::ForkManager\" module

#In ORFcor directory

ls ~/MollicuteGenomes2021/Feb8MesoORFcor/ORFcor_multiple_sequence_files/*.faa > CorrectedFeb8TestMesoFastaList

#In ~/MollicuteGenomes2021/Feb8MesoORFcor Directory

perl ~/ORFcor/Mult_MUSCLE.pl -i ~/ORFcor/CorrectedFeb8TestMesoFastaList

#6) Concatenates multiple alignments, and creates partitions file for RAxML (one partition per protein type). Removes columns comprised of gaps > than some threshold.

Aligned_multiple_faa_concatenator.pl

#In ~/MollicuteGenomes2021/Feb8MesoORFcor Directory

perl ~/ORFcor/Aligned_multiple_faa_concatenator.pl -i Mult_MUSCLE_corrected_multiple_faas

conda activate anvio-7

FastTree -wag ~/MollicuteGenomes2021/Feb8MesoORFcor/Aligned_multiple_faa_concatenator.faa > JKScript_Feb8Test_Core_Bin_1_SeqsPhylog.tree

#It worked!! Put tree into ITol

#Now to figure out nucleotides...

anvi-get-sequences-for-gene-clusters -p Split_Feb8Test_Pans/Core_Bin_1/PAN.db -g Feb8Test-GENOMES.db -o Feb8Test_Core_Bin_1_NuclPhylog.fa --report-DNA-sequences --max-num-genes-from-each-genome 1 --just-do-it

perl Convert_anvio_mult_faa_to_family_faas.pl -i Feb8Test_Core_Bin_1_NuclPhylog.fa -o Feb8TestMesoNuclOutput

#1) #In ORFcore directory:

ls ~/MollicuteGenomes2021/Feb8TestMesoNuclOutput/*.faa > Feb8TestMesoNuclFastaList

#4) #made Feb8MesoNuclORFcor directory to keep everything together

Run in ~/MollicuteGenomes2021/Feb8MesoNuclORFcor

perl ~/ORFcor/ORFcor.pl -i ~/ORFcor/Feb8TestMesoNuclFastaList

#made the directory ORFcor_multiple_sequence_files

#5) #In ORFcor directory

ls ~/MollicuteGenomes2021/Feb8MesoNuclORFcor/ORFcor_multiple_sequence_files/*.faa > CorrectedFeb8TestMesoNuclFastaList

#In ~/MollicuteGenomes2021/Feb8MesoNuclORFcor Directory

perl ~/ORFcor/Mult_MUSCLE.pl -i ~/ORFcor/CorrectedFeb8TestMesoNuclFastaList

6)#In ~/MollicuteGenomes2021/Feb8MesoNuclORFcor Directory

perl ~/ORFcor/Aligned_multiple_faa_concatenator.pl -i Mult_MUSCLE_corrected_multiple_faas

conda activate anvio-7

FastTree -nt ~/MollicuteGenomes2021/Feb8MesoNuclORFcor/Aligned_multiple_faa_concatenator.faa > JKScript_Feb8Test_Core_Bin_1_NuclPhylog.tree

#Spiro

#IN SpiroplasmaGenomes DIRECTORY

#Feb 15 2022

anvi-gen-genomes-storage -i InternalSpiro.txt -e ExternalGenomesPlatyhelixEntAcro10.txt -o Feb15Spiro-GENOMES.db --gene-caller 'Prodigal'

anvi-pan-genome -g Feb15Spiro-GENOMES.db -n Feb15Spiro_anvipan --output-dir Feb15Spiro_AnviPan --num-threads 10 --minbit 0.5 --mcl-inflation 3

# mcl-inflation to 3

anvi-display-pan -p Feb15Spiro_AnviPan/Feb15Spiro_anvipan-PAN.db -g Feb15Spiro-GENOMES.db

#Min number of genomes gene cluster occurs= 3 & Max functional homogeneity index=.99 684 gene clusters

#Feb15SpiroState

#Feb15SpiroBins

#Core_Bin

anvi-split -p Feb15Spiro_AnviPan/Feb15Spiro_anvipan-PAN.db -g Feb15Spiro-GENOMES.db -C Feb15SpiroBins -o Feb15Split_Spiro_Pans

#Don't Concatenate gene clusters

anvi-get-sequences-for-gene-clusters -p Feb15Split_Spiro_Pans/Core_Bin/PAN.db -g Feb15Spiro-GENOMES.db -o Feb15SpiroCore_Bin_SeqsPhylog.fa --max-num-genes-from-each-genome 1

#Genes clusters that failed the filter (44) ..................: GC_00000012, GC_00000013, GC_00000003, (... 41 more (`--debug` will show all))

#Feb222022

In ~/MollicuteGenomes2021/SpiroplasmaGenomes/

perl Convert_anvio_mult_faa_to_family_faas.pl -i Feb15SpiroCore_Bin_SeqsPhylog.fa -o Feb15SpiroFaasOutput

1) #In ORFcore directory:

ls ~/MollicuteGenomes2021/SpiroplasmaGenomes/Feb15SpiroFaasOutput/*.faa > Feb15SpiroFastaList

4) #made Feb15SpiroORFcor directory to keep everything together

Run in ~/MollicuteGenomes2021/SpiroplasmaGenomes/Feb15SpiroORFcor

perl ~/ORFcor/ORFcor.pl -i ~/ORFcor/Feb15SpiroFastaList

#made the directory ORFcor_multiple_sequence_files

5) #In ORFcor directory

ls ~/MollicuteGenomes2021/SpiroplasmaGenomes/Feb15SpiroORFcor/ORFcor_multiple_sequence_files/*.faa > CorrectedFeb15SpiroFastaList

#In ~/MollicuteGenomes2021/SpiroplasmaGenomes/Feb15SpiroORFcor Directory

perl ~/ORFcor/Mult_MUSCLE.pl -i ~/ORFcor/CorrectedFeb15SpiroFastaList

6)#In ~/MollicuteGenomes2021/SpiroplasmaGenomes/Feb15SpiroORFcor Directory

perl ~/ORFcor/Aligned_multiple_faa_concatenator.pl -i Mult_MUSCLE_corrected_multiple_faas

conda activate anvio-7

FastTree -wag ~/MollicuteGenomes2021/SpiroplasmaGenomes/Feb15SpiroORFcor/Aligned_multiple_faa_concatenator.faa > JKScript_Feb15SpiroCore_Bin_SeqsPhylog.tree

#IT WORKED! Put tree into ITol

#Now to figure out nucleotides...

anvi-get-sequences-for-gene-clusters -p Feb15Split_Spiro_Pans/Core_Bin/PAN.db -g Feb15Spiro-GENOMES.db -o Feb15SpiroCore_Bin_NuclPhylog.fa --report-DNA-sequences --max-num-genes-from-each-genome 1 --just-do-it

In ~/MollicuteGenomes2021/SpiroplasmaGenomes/

perl Convert_anvio_mult_faa_to_family_faas.pl -i Feb15SpiroCore_Bin_NuclPhylog.fa -o Feb15SpiroNuclOutput

1) #In ORFcore directory:

ls ~/MollicuteGenomes2021/SpiroplasmaGenomes/Feb15SpiroNuclOutput/*.faa > Feb15SpiroNuclFastaList

4) #made Feb15SpiroNuclORFcor directory to keep everything together

Run in ~/MollicuteGenomes2021/SpiroplasmaGenomes/Feb15SpiroNuclORFcor

perl ~/ORFcor/ORFcor.pl -i ~/ORFcor/Feb15SpiroNuclFastaList

#made the directory ORFcor_multiple_sequence_files

5) #In ORFcor directory

ls ~/MollicuteGenomes2021/SpiroplasmaGenomes/Feb15SpiroNuclORFcor/ORFcor_multiple_sequence_files/*.faa > CorrectedFeb15SpiroNuclFastaList

#In ~/MollicuteGenomes2021/SpiroplasmaGenomes/Feb15SpiroNuclORFcor Directory

perl ~/ORFcor/Mult_MUSCLE.pl -i ~/ORFcor/CorrectedFeb15SpiroNuclFastaList

6)#In ~/MollicuteGenomes2021/SpiroplasmaGenomes/Feb15SpiroNuclORFcor Directory

perl ~/ORFcor/Aligned_multiple_faa_concatenator.pl -i Mult_MUSCLE_corrected_multiple_faas

conda activate anvio-7

FastTree -nt ~/MollicuteGenomes2021/SpiroplasmaGenomes/Feb15SpiroNuclORFcor/Aligned_multiple_faa_concatenator.faa > JKScript_Feb15SpiroCore_Core_Bin_NuclPhylog.tree

Convert_anvio_mult_faa_to_family_faas.pl

#!/usr/bin/perl

#

# Jonathan Klassen

# v1.1 February 21, 2022

#

# Convers the multiple faa file produced by anvio containing proteins from all families

# into individual faa files each containing only proteins from a single family.

# These files can then be fed to ORFcor for alignment and analysis.

use strict;

use warnings;

use Getopt::Long;

############################################################################

# Processes input arguments

############################################################################

my $use = "Convert_anvio_mult_faa_to_family_faas.pl

DEPENDANCIES: none

USE:

-h displays this usage statement (also using --help)

-i input sqn file whose numbers won't be changed REQUIRED e.g., perl Convert_anvio_mult_faa_to_family_faas.pl -i all_prots.faa

-o folder that contains the output faas DEFAULT: output e.g., perl Convert_anvio_mult_faa_to_family_faas.pl -i all_prots.faa -o new_faas

-q run quietly, i.e., no STDOUT (Y or N) DEFAULT: N e.g., perl Convert_anvio_mult_faa_to_family_faas.pl --i all_prots.faa -q Y

OUTPUT FILES:

a folder containing individual faa files (specified by -o), each named according to their anvio protein family name

";

# input arguements

my %options = ();

GetOptions(

"h" => \$options{help},

"help" => \$options{help},

"i=s" => \$options{infile},

"o=s" => \$options{outfolder},

"q=s" => \$options{quiet},

);

# display use statement if called

die $use if ($options{help});

# defaut arguments

unless ($options{outfolder}){ $options{outfolder} = "output"};

unless ($options{quiet}){ $options{quiet} = "N"};

# mystery input flags not allowed

die "Unrecognized command line arguments: @ARGV\n" if ($ARGV[0]);

# checks correct parameter formatting

die "Unrecognized command line arguements: -q = $options{quiet}\n$use" unless ($options{quiet} eq "Y" or $options{quiet} eq "N");

# required arguements

die "Input file -i is not specified:\n, $use" if (!$options{infile});

my $infile1_name = $options{infile};

# print parameters unless -q flag selected

print "-----------------------------------------------------------------------------

Convert_anvio_mult_faa_to_family_faas.pl Jonathan Klassen v1.1 February 21, 2022

parameters used:

input file = $options{infile}

output folder = $options{outfolder}

quiet = $options{quiet}

-----------------------------------------------------------------------------

" if ($options{quiet} eq "N");

#############################################################################

# Loads input file

#############################################################################

open (INFILE, $infile1_name) or die "Cannot open $infile1_name\n\n$use";

unless(-d $options{outfolder}){ mkdir($options{outfolder}) }

my @infile = <INFILE>;

close INFILE;

my %families;

my $gene_cluster = my $header = my $strain = my $gene = '';

foreach my $line (@infile){

if ($line =~ /^>/){

$line =~ /^>(\w+)\|gene_cluster:(\w+)\|genome_name:(\w+)/ or die "Cannot match $line";

$gene = $1;

$gene_cluster = $2;

$strain = $3;

$header = ">$gene $strain\n";

}

else {

$families{$gene_cluster}{$header} = $line;

}

}

#############################################################################

# Creates output files

#############################################################################

foreach my $gene_cluster (sort keys %families){

open (OUTFILE, ">>$options{outfolder}/$gene_cluster.faa")

or die "Cannot open $options{outfolder}/$gene_cluster.faa";

foreach my $header (sort keys %{$families{$gene_cluster}}){

print OUTFILE $header;

print OUTFILE $families{$gene_cluster}{$header};

}

close OUTFILE;

}
