# Supplementary material for: Isolation and characterization of mollicute symbionts from a fungus-growing ant reveals high niche overlap leading to co-exclusion
Source: mBio. 2025 Jun 10;16(7):e00893-25. doi: 10.1128/mbio.00893-25 (PMC12239587; doi:10.1128/mbio.00893-25)
Supplement: Supplemental Material — Supplemental figures and tables. [file mbio.00893-25-s0002.pdf]

Supplementary figures

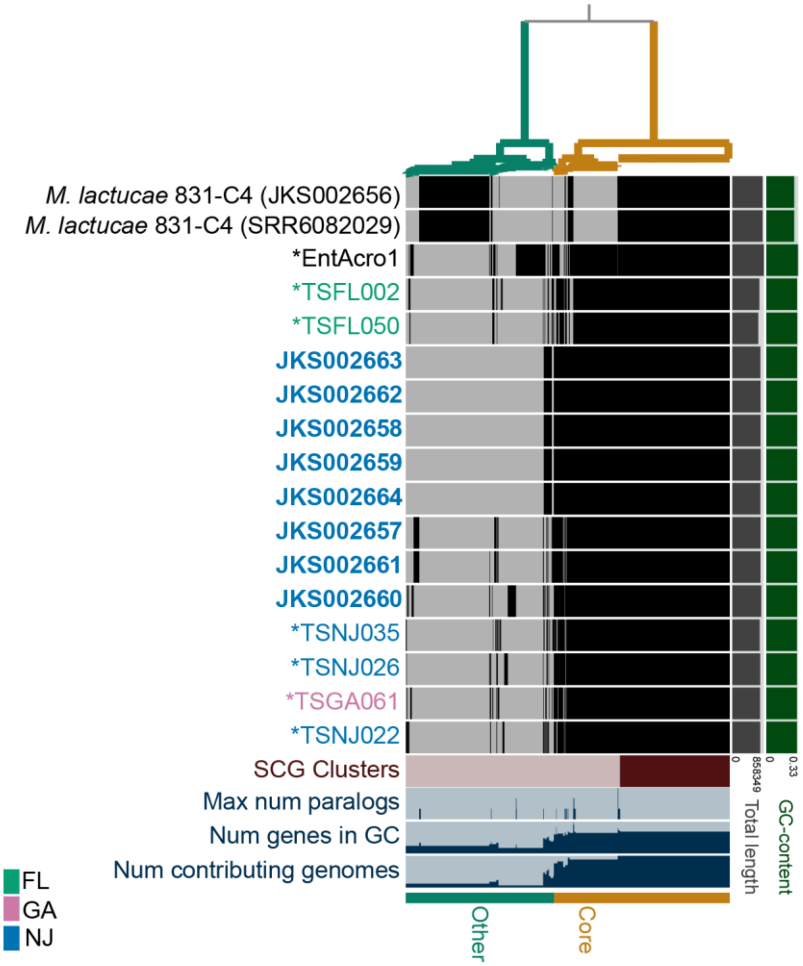

Supplementary Figure S1) Anvi'o pangenome analysis of *M. whartonense* genomes (bold) and MAGs with a 100% BUSCO score (*), the EntoAcro1 MAG (*), and the *M. lactucae* genomes as a reference. Text colors indicate strains or MAGs from the same state. Two *M. lactucae* genomes are shown, representing the genome downloaded from NCBI (SRR6082029) and the genome that we re-sequenced as a control (JKS002656). In the central heatmap, black and grey bars indicate homologous genes that are present or absent in each genome. The dendrogram clusters groups of homologous genes (anvi'o gene clusters) using Euclidean distances.

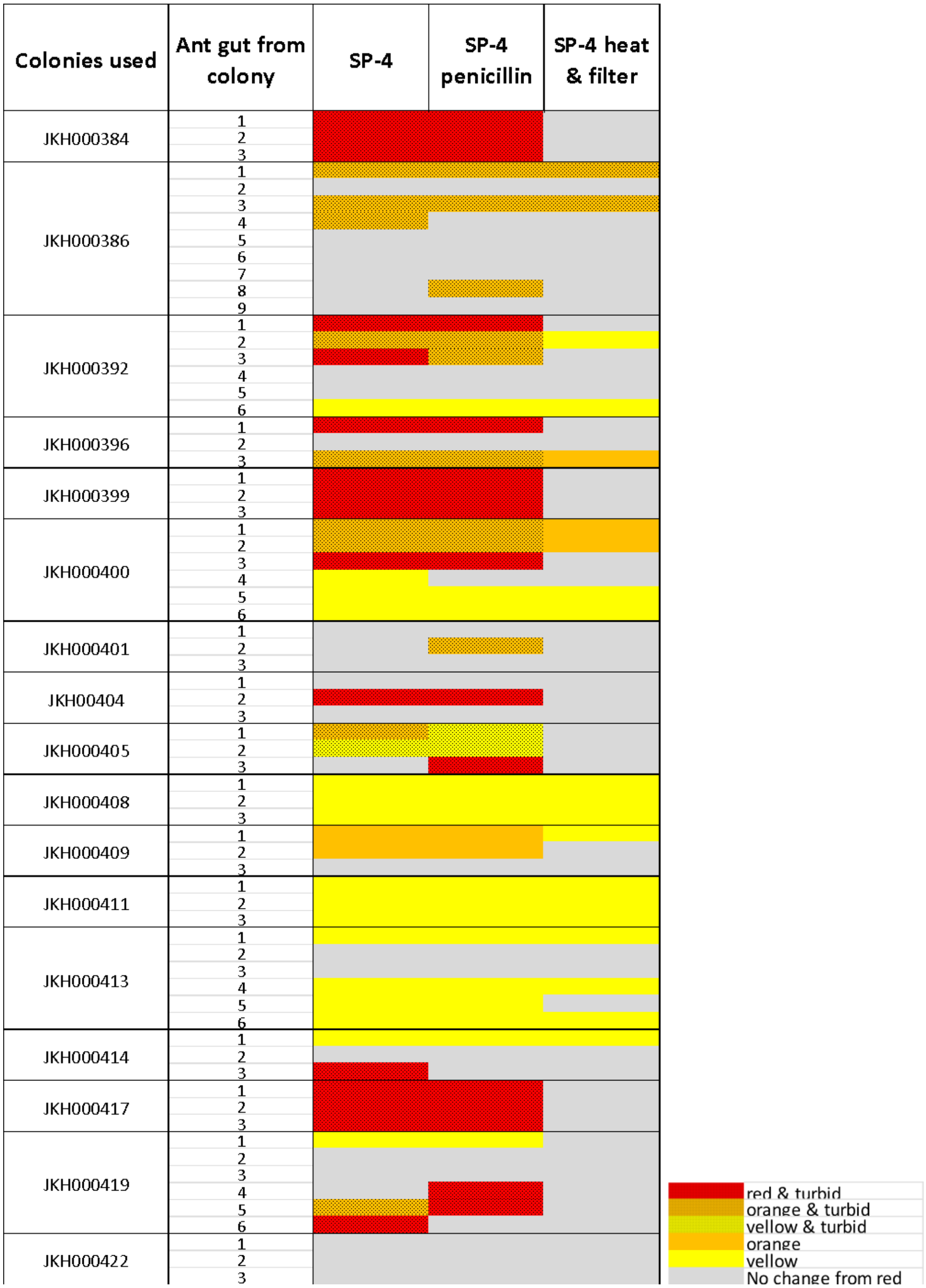

Supplementary Figure S2) Heatmap describing the cultures used to enrich *Mesoplasma* and *Spiroplasma* from ant guts.

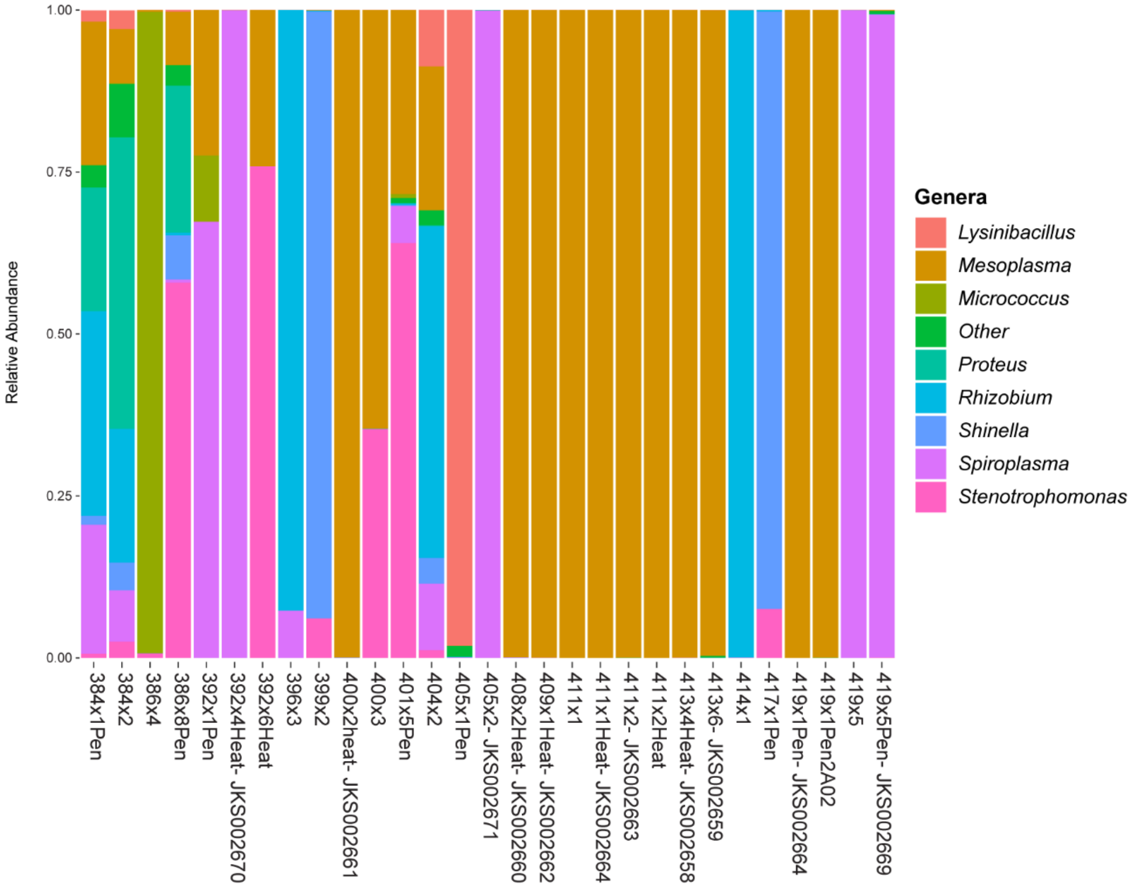

Supplementary Figure S3) Bar plot representing the top genera present (≥ 15% abundance) in our 16S rRNA community amplicon sequencing of cultures. Each bar represents an individual culture. On the x-axis, names with a JKS prefix indicate samples that were used isolation and genome sequencing.

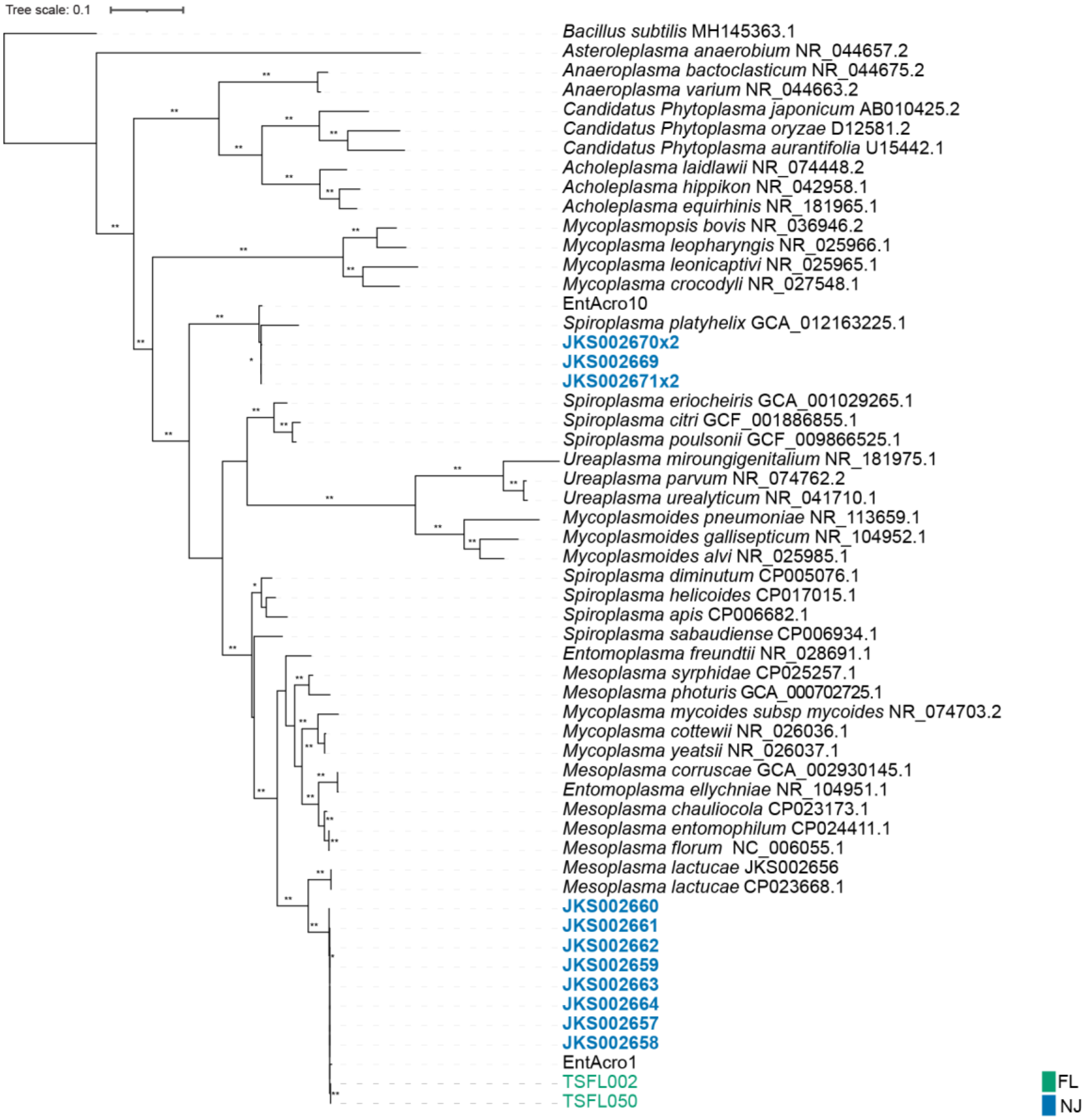

Supplemental Figure S4) Mollicute phylogeny created using full-length 16S rRNA genes. Asterisks indicate sequences from MAGs and bolded names indicate sequences from *M. whartonense* and *S. attinicola* isolate genomes. Blue text indicates strains isolated from New Jersey and green text indicates MAGs from Florida. The tree was rooted using *Bacillus subtilis*. Local bootstrap values of 60–79 and 80–100 are indicated by * and **, respectively. Note that low bootstrap support disrupts the monophyly of the genus *Spiroplasma* in this tree, likely indicating the insufficient phylogenetic resolution of the 16S rRNA gene for resolving these

nodes. Nevertheless, all ant-associated *Spiroplasma* strains are most closely related to *S. platyhelix* in this phylogeny with high bootstrap support.

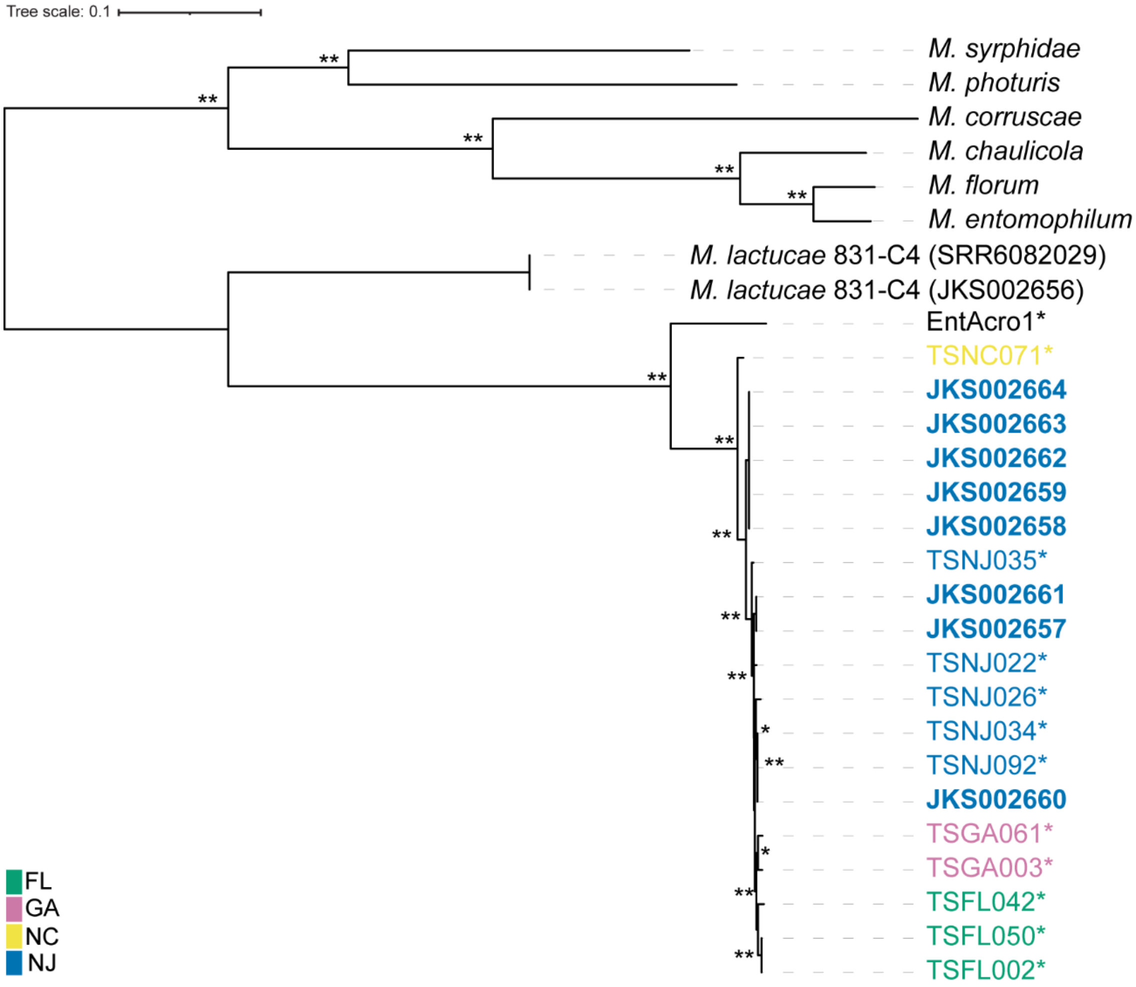

Supplementary Figure S5) *Mesoplasma* amino acid phylogeny created using 21 single-copy core gene clusters as input data and the FastTree WAG + CAT substitution model. Asterisks indicate MAGs and bolded names indicate *M. whartonense* isolate genomes. Colors group strains and MAGs by state. The tree was rooted using the midpoint of the branch between *M. lactucae* and the other reference genomes. Local bootstrap values of 60–79 and 80–100 are indicated by * and **, respectively.

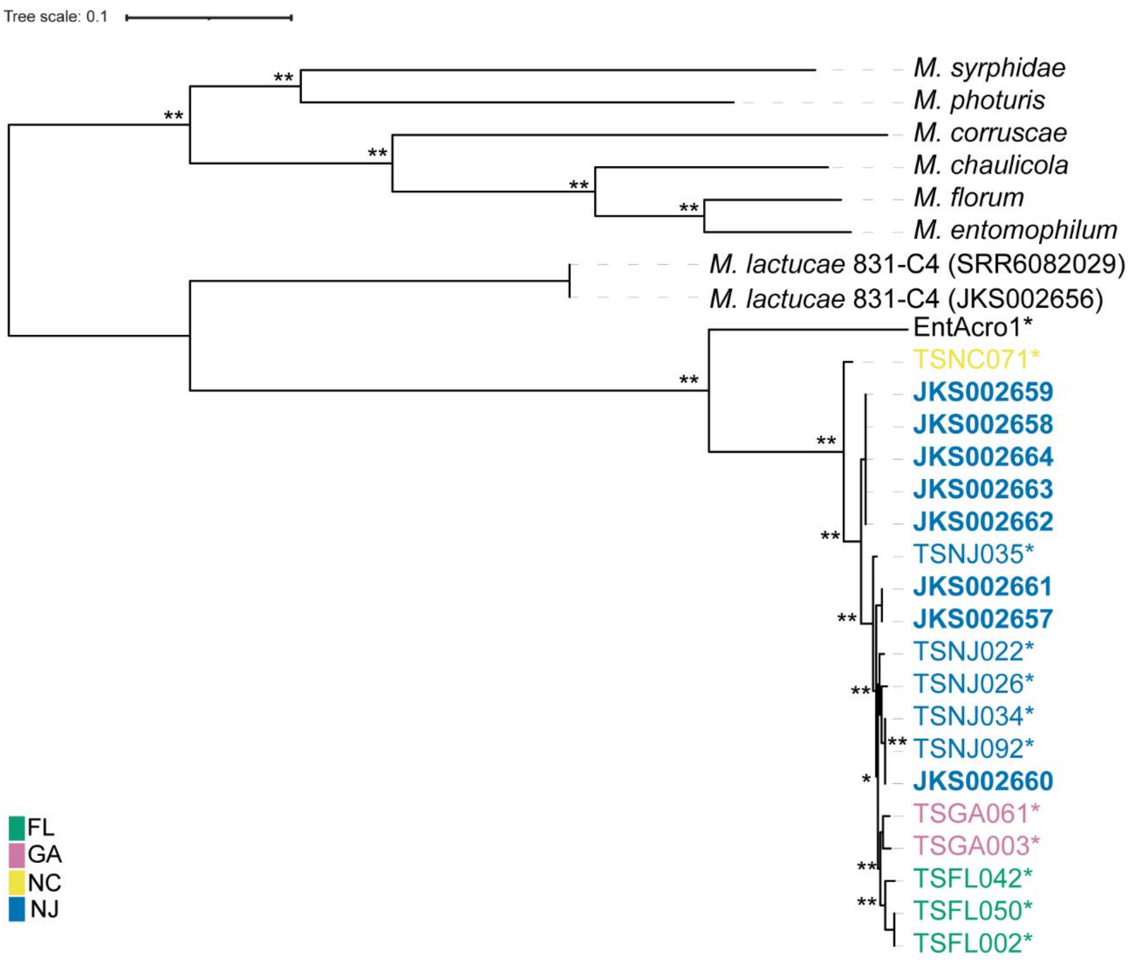

Supplementary Figure S6) *Mesoplasma* nucleotide phylogeny created using 21 single-copy core gene clusters as input data and the FastTree JC + CAT substitution model. Asterisks indicate MAGs and bolded names indicate *M. whartonense* isolate genomes. Colors group strains and MAGs by state. The tree was rooted using the midpoint of the branch between *M. lactucae* and the other reference genomes. Local bootstrap values of 60–79 and 80–100 are indicated by * and **, respectively.

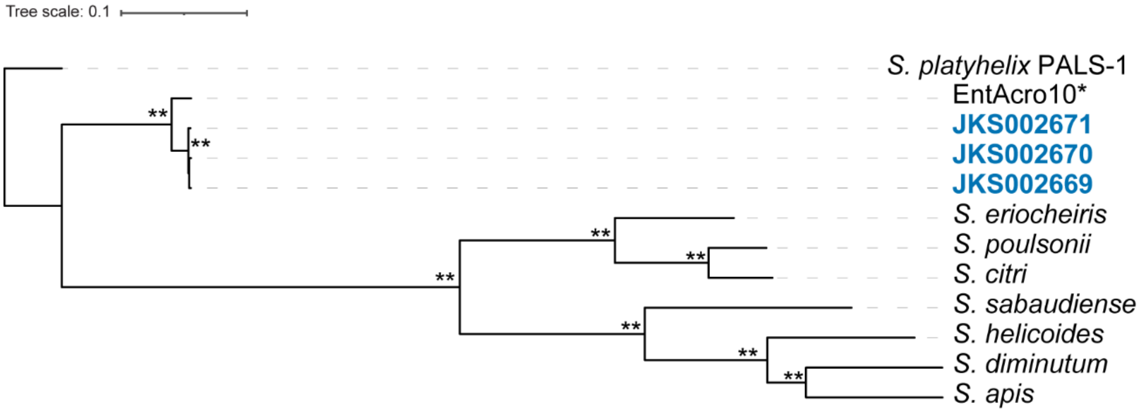

Supplementary Figure S7) *Spiroplasma* amino acid phylogeny created using 80 single-copy core gene clusters as input data and the FastTree WAG + CAT substitution model. Asterisks indicate MAGs and bolded names indicate *S. attinicola* isolate genomes. The blue color indicates strains from New Jersey. The tree was rooted using the *S. platyhelix* genome. Local bootstrap values of 60–79 and 80–100 are indicated by * and **, respectively.

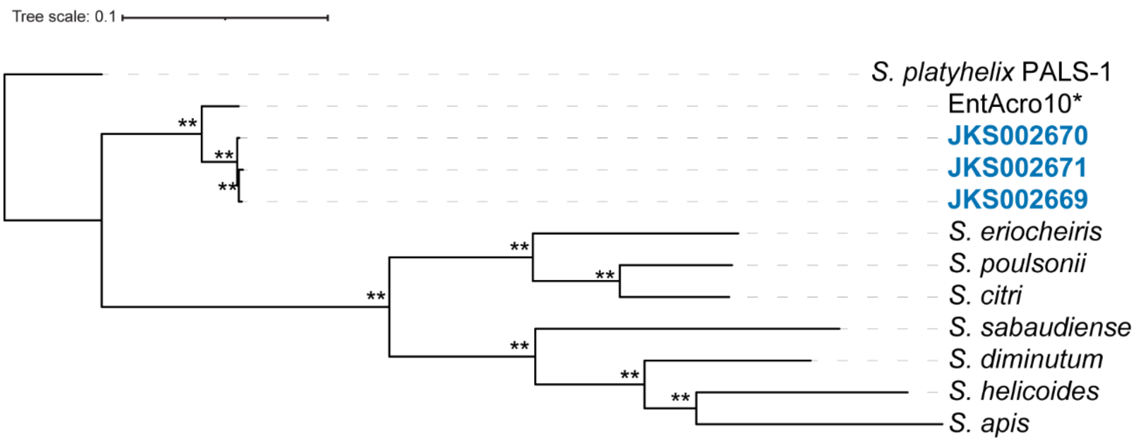

Supplementary Figure S8) *Spiroplasma* nucleotide phylogeny created using 80 single-copy core gene clusters as input data and the FastTree JC + CAT substitution model. Asterisks indicate MAGs and bolded names indicate *S. attinicola* isolate genomes. The blue color indicates strains from New Jersey. The tree was rooted using the *S. platyhelix* genomes. Local bootstrap values of 60–79 and 80–100 are indicated by * and **, respectively.

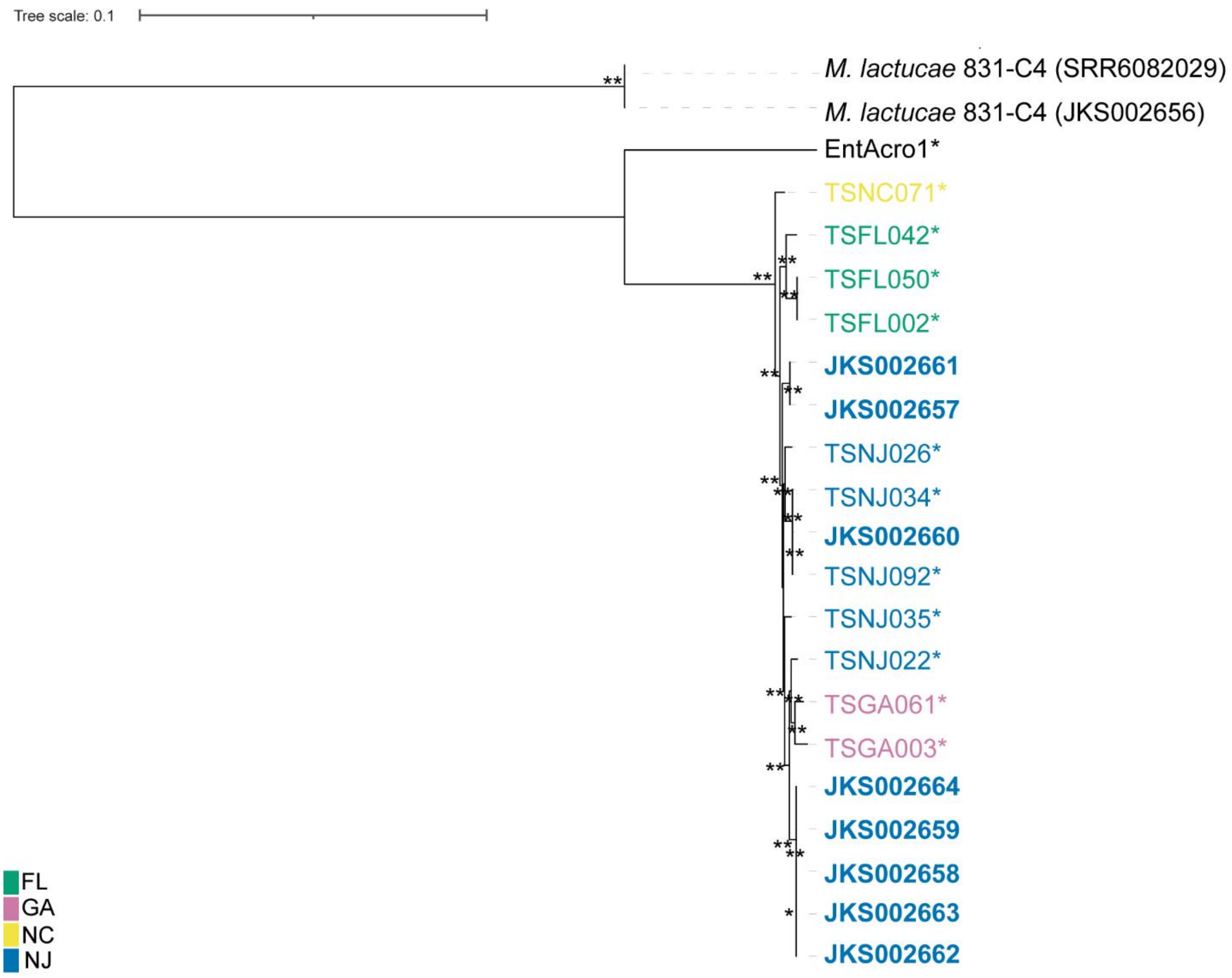

Supplementary Figure S9) *Mesoplasma* amino acid phylogeny created using 550 core gene clusters as input data and the FastTree WAG + CAT substitution model. Asterisks indicate MAGs and bolded names indicate *M. whartonense* isolate genomes. Colors group strains and MAGs by state. The tree was rooted using the midpoint of the branch between *M. lactucae* genomes. Local bootstrap values of 60–79 and 80–100 are indicated by * and **, respectively.

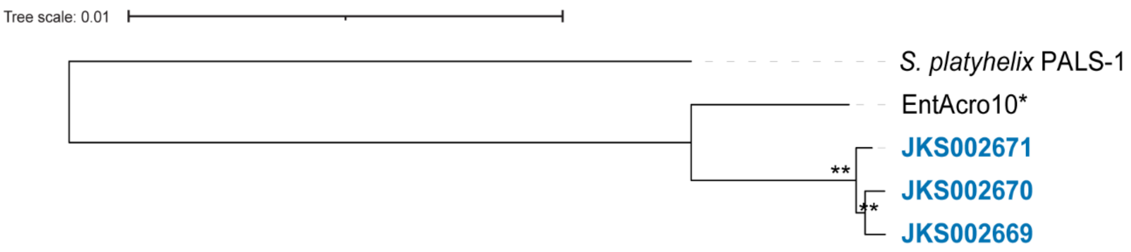

Supplementary Figure S10) *Spiroplasma* amino acid phylogeny created using 684 core gene clusters as input data and the fastree WAG + CAT substitution model. Asterisks indicate MAGs and bolded names indicate *S. attinicola* isolate genomes, with the blue text indicating strains isolated from New Jersey. The tree was rooted using the *S. platyhelix* genome. Local bootstrap values of 60–79 and 80–100 are indicated by * and **, respectively.

Supplemental Tables

Supplementary Table S1) Colony ID, date and location with the respective Genome or MAG ID associated with that colony.

Colony ID	Collection Date	Geographic Location	GPS Latitude	GPS Longitude	Genome or MAG ID
JKH000014	5/20/2013	Florida:Wekiwa Springs State Park	28.70991	-81.46795	TSFL002
JKH000065	7/22/2014	New Jersey:Brendon T. Byrne State Forest	39.87275	-74.52157	TSNJ022
JKH000069	7/22/2014	New Jersey:Brendon T. Byrne State Forest	39.87212	-74.52075	TSNJ026
JKH000073	7/23/2014	New Jersey:Wharton State Forest	39.71267	-74.56350	TSNJ092
JKH000079	7/23/2014	New Jersey:Wharton State Forest	39.71275	-74.56442	TSNJ034
JKH000082	7/24/2014	New Jersey:Wharton State Forest:Goshen pond	39.74587	-74.76150	TSNJ035
JKH000105	11/14/2014	Florida:Wekiwa Springs	28.70900	-81.48730	TSFL042
JKH000119	11/16/2014	Florida:Paynes	27.62286	-81.80370	TSFL050
JKH000136	5/7/2015	Geogia:Yuchi Wildlife Management Area	33.08388	-81.77029	TSGA003
JKH000138	5/7/2015	Geogia:Yuchi Wildlife Management Area	33.08393	-81.76996	TSGA061
JKH000154	6/9/2015	North Carolina:William B Umpstead State Park	35.86070	-78.76103	TSNC071
JKH000392	6/23/2020	New Jersey:Wharton State Forest:Quaker Bridge	39.70921	-74.66321	JKS002670
JKH000400	6/24/2020	New Jersey:Wharton State Forest:Batona Field	39.78143	-74.62846	JKS002661
JKH000405	6/24/2020	New Jersey:Wharton State Forest:Batona Field	39.78159	-74.62822	JKS002671
JKH000408	6/25/2020	New Jersey:Wharton State Forest:Hawkins Bridge	39.71257	-74.56377	JKS002660
JKH000409	6/25/2020	New Jersey:Wharton State Forest:Hawkins Bridge	39.71259	-74.56346	JKS002662
JKH000411	6/25/2020	New Jersey:Wharton State Forest:Hawkins Bridge	39.71284	-74.56367	JKS002663 & JKS002664
JKH000413	6/25/2020	New Jersey:Wharton State Forest:Hawkins Bridge	39.71269	-74.56355	JKS002658 & JKS002659
JKH000419	6/26/2020	New Jersey:Brendan Byrne State Forest	39.87226	-74.52554	JKS002657 & JKS002669

Supplementary Table S2) Characteristics of the *T. septentrionalis Mesoplasma* MAGs sequenced by JGI.

Name	Colony ID	Length (bp)	BUSCO completeness score	Contigs	% GC	JGI Bin ID
TSFL002	JKH000014	738626	94.70%	45	32.72%	3300012079_26
TSFL042	JKH000105	615878	67.30%	36	32.41%	3300012141_10
TSFL050	JKH000119	738262	94.70%	45	32.72%	3300012098_5
TSGA003	JKH000136	524149	72%	20	32.56%	3300012101_6
TSGA061	JKH000138	752725	100%	9	32.62%	3300012067_9
TSNC071	JKH000154	642949	82%	8	32.58%	3300011392_7
TSNJ022	JKH000065	758990	100%	10	32.51%	3300012151_17
TSNJ026	JKH000069	760750	100%	9	32.55%	3300012165_16
TSNJ034	JKH000079	385824	52.70%	77	33%	3300012155_29
TSNJ035	JKH000082	744761	100%	9	32.57%	3300011404_8
TSNJ092	JKH000073	612061	69.30%	9	32.55%	3300012100_6

Supplementary Table S3) Characteristics of the *Mesoplasma* and *Spiroplasma* MAGs published by Sapountzis et al. [39]

Name	Length (bp)	BUSCO completeness score	Contigs	% GC
EntAcro1	866917	100%	16	33.74%
EntAcro10	838545	98%	13	25.53%

Supplementary Table S4) Characteristics of the *Mesoplasma* and *Spiroplasma* reference genomes downloaded from NCBI.

**Name**	**Strain**	**ATCC ID #**	**Assembly**	**BioSample**	**BioProject**	**Size (bp)**
Mesoplasma florum	L1	ATCC:33453	GCF_000008305.1	SAMN03081416	PRJNA224116	793,224
Mesoplasma corruscae	ELCA-2		GCA_002930145.1	SAMN05436728	PRJNA331058	839,085
Mesoplasma chauliocola	CHPA-2		GCF_002290085.1	SAMN07573570	PRJNA224116	854,780
Mesoplasma photuris		ATCC:49581	GCA_000702725.1	SAMN02841188	PRJNA223039	778,966
Mesoplasma syrphidae	YJS	ATCC:51578	GCF_002843565.1	SAMN08158127	PRJNA224116	908,214
Mesoplasma entomophilum	TAC		GCF_002749675.1	SAMN07838951	PRJNA224116	847,967
Mesoplasma lactucae	831-C4	ATCC:49193	GCA_002441935.1	SAMN07709945	PRJNA412357	837,471
Spiroplasma citri	R8-A2		GCF_001886855.1	SAMN04110376	PRJNA296877	1,599,709
Spiroplasma eriocheiris	DSM 21848		GCA_001029265.1	SAMN03488050	PRJNA253647	1,365,714
Spiroplasma sabaudiense	Ar-1343		GCA_000565215.1	SAMN03081504	PRJNA184750	1,075,953
Spiroplasma poulsonii	MSRO_BK		GCF_009866525.1	SAMN11349289	PRJNA224116	1,938,611
Spiroplasma apis	B31	ATCC:33834	GCA_000500935.1	SAMN02641479	PRJNA184751	1,160,554
Spiroplasma diminutum	CUAS-1		GCA_000439455.1	SAMN02602967	PRJNA184745	945,296
Spiroplasma helicoides	TABS-2		GCA_001715535.1	SAMN05578878	PRJNA253649	1,326,546
Spiroplasma platyhelix	PALS-1	ATCC 51748	GCA_012163225.1	SAMN14173566	PRJNA608455	739,863

Supplementary Table S5) Characteristics of the *M. whartonense* and *S. attinicola* isolate genomes and *M. lactucae* reference genomes sequenced in this study.

	Strain ID	Sample ID	Colony ID	Contigs before filtering	Contigs after filtering	% GC	BUSCO complete-ness score[a]	Largest contig (bp)	Total length (bp)	NCBI WGS accession
Mesoplasma	JKS002656			8	8	29.63	99.30%	443,977	824,849	JAKNSY000000000
	JKS002657		JKH000419	8	5	32.74	100%	308,895	769,567	JAKNSX000000000
	JKS002658	JKA007839	JKH000413	12	7	32.79	100%	448,687	767,909	JAKNSW000000000
	JKS002659	JKA007841	JKH000413	10	6	32.79	100%	448,687	770,321	JAKNSV000000000
	JKS002660	JKA007836	JKH000408	8	6	32.68	100%	545,137	786,541	JAKNSU000000000
	JKS002661	JKA007851	JKH000400	6	5	32.74	100%	284,762	769,577	JAKNST000000000
	JKS002662	JKA007834	JKH000409	12	7	32.79	100%	448,689	767,977	JAKNSS000000000
	JKS002663	JKA007853	JKH000411	14	9	32.70	100%	221,966	762,874	JAKNSR000000000
	JKS002664	JKA007852	JKH000411	13	9	32.72	100%	221,966	765,176	JAKNSQ000000000
Spiroplasma	JKS002669	JKA007833	JKH000419	12	4	25.7	98.70%	632,388	835,789	JAKQXY000000000
	JKS002670	JKA007855	JKH000392	18	8	25.49	98.70%	372,052	872,740	JAKNSP000000000
	JKS002671	JKA007849	JKH000405	10	5	25.77	98.70%	528,435	884,092	JAKNSO000000000

[a]BUSCO scores were determined for the filtered contigs

Supplementary Table S6) Classic RAST Overview of the *M. whartonense* and *S. attinicola* genomes, *Mesoplasma* MAGs, EntAcro MAGs, and the *M. lactucae* and *S. platyhelix* reference genomes.

	Mesoplasma														*Spiroplasma*				
**RAST Annotation**	**EnAcro1**	**JKS002657**	**JKS002658**	**JKS002659**	**JKS002660**	**JKS002661**	**JKS002662**	**JKS002663**	**JKS002664**	**TSGA061**	**TSNJ022**	**TSNJ026**	***M. lactucae***	**TSNJ035**	**EntAcro10**	**JKS002669**	**JKS002670**	**JKS002671**	***S. platyhelix***
Number of Subsystems	152	147	151	151	152	147	151	152	152	148	151	150	148	148	141	139	140	140	135
Number of Coding Sequences	757	671	661	666	689	668	662	660	663	655	663	663	691	654	774	768	820	814	670
Number of RNAs	35	34	37	37	34	34	37	34	34	35	33	33	35	33	29	29	29	29	29
Cofactors, Vitamins, Prosthetic Groups, Pigments	42	43	43	42	43	43	43	43	43	43	44	43	48	43	46	35	35	35	24
Cell Wall and Capsule	4	4	4	4	4	4	4	4	4	4	4	4	4	4	2	2	2	2	2
Virulence, Disease and Defense	16	16	20	19	20	16	20	20	20	16	21	20	19	20	23	18	18	18	17
Potassium metabolism	6	2	2	2	2	2	2	2	2	2	2	2	6	2	2	2	2	2	2
Photosynthesis	0	0	0	0	0	0	0	0	0	0	0	0	0	0	0	0	0	0	0
Miscellaneous	1	1	1	1	1	1	1	1	1	1	1	1	1	1	1	1	1	1	1
Phages, Prophages, Transposable elements, Plasmids	0	0	0	0	0	0	0	0	0	0	0	0	0	0	0	0	0	0	0
Membrane Transport	19	20	20	20	20	20	20	20	20	20	20	20	22	20	13	14	12	15	15
Iron acquisition and metabolism	0	0	0	0	0	0	0	0	0	0	0	0	0	0	0	0	0	0	0
RNA Metabolism	50	49	49	49	49	49	49	49	49	49	49	49	45	49	47	46	46	46	44
Nucleosides and Nucleotides	27	27	27	27	27	27	27	27	27	26	27	27	20	26	28	29	29	29	28
Protein Metabolism	137	117	117	139	139	117	117	139	139	139	139	139	143	117	132	134	133	132	132
Cell Division and Cell Cycle	11	12	12	12	12	12	12	12	12	12	12	12	9	12	15	15	15	15	15
Motility and Chemotaxis	0	0	0	0	0	0	0	0	0	0	0	0	0	0	0	0	0	0	0
Regulation and Cell signaling	3	3	3	3	3	3	3	3	3	3	3	3	3	3	2	2	2	2	2
Secondary Metabolism	0	0	3	3	3	0	3	3	3	0	3	3	0	3	0	0	0	0	0
DNA Metabolism	45	39	50	48	48	39	50	50	50	42	44	41	40	36	38	42	42	44	35
Fatty Acids, Lipids, and Isoprenoids	12	12	12	12	12	12	12	12	12	12	12	12	14	12	3	3	3	3	5
Nitrogen Metabolism	0	0	0	0	0	0	0	0	0	0	0	0	0	0	0	0	0	0	0
Dormancy and Sporulation	1	1	1	1	1	1	1	1	1	1	1	1	1	1	1	1	1	1	1
Respiration	8	8	8	8	8	8	8	8	8	8	8	8	9	8	3	3	3	3	9
Stress Response	10	10	19	10	10	10	10	10	10	10	10	10	12	10	4	3	3	12	3
Metabolism of Aromatic Compounds	0	0	9	0	0	0	0	0	0	0	0	0	0	0	0	0	0	0	0
Amino Acids and Derivatives	21	22	24	24	23	22	24	24	24	23	23	23	6	23	20	20	20	20	17
Sulfur Metabolism	2	2	2	2	2	2	2	2	2	2	2	2	3	2	2	1	1	1	1
Phosphorus Metabolism	11	11	11	11	11	11	11	11	11	11	11	11	11	11	5	5	5	5	6
Carbohydrates	46	47	46	47	46	46	46	46	47	46	48	48	43	46	49	47	54	47	50

Supplementary Table S7) *Mesoplasma* and *Spiroplasma* ASV matches to our Mollicute isolate genomes. The most abundant ASV from both the ant microbiome study [35] and the ant gut enrichment cultures (Suppl. Fig. S3) was used as a query sequences.

	**Query**	**Reference**	**% Identity**	**Query coverage**
**Ant Microbiome**	*Mesoplasma* ASV	JKS002658 genome	100	100
	Spiroplasma ASV	JKS002669 genome	100	100
**Ant Gut Cultures**	*Mesoplasma* ASV	JKS002658 genome	100	100
	Spiroplasma ASV	JKS002669 genome	100	100
